# Synthesis, X-ray crystallography, spectroscopic characterizations, and density functional theory of the chloride-bound five-coordinate high-spin Iron(II) “Picket Fence” porphyrin complex

**DOI:** 10.3389/fchem.2025.1607585

**Published:** 2025-07-14

**Authors:** Feriel Salhi, Mondher Dhifet, Bouzid Gassoumi, Noureddine Issaoui, Habib Nasri

**Affiliations:** ^1^ Laboratory of Physical Chemistry of Materials (LR01ES19), Faculty of Sciences of Monastir, Monastir, Tunisia; ^2^ Faculty of Sciences of Gafsa, University of Gafsa, Gafsa, Tunisia; ^3^ Laboratory of Advanced Materials and Interfaces (LIMA), Faculty of Sciences of Monastir, University of Monastir, Monastir, Tunisia; ^4^ Laboratory of Quantum and Statistical Physics LR18ES18, Faculty of Sciences of Monastir, University of Monastir, Monastir, Tunisia; ^5^ Higher Institute of Computer Sciences and Mathematics of Monastir, University of Monastir, Monastir, Tunisia

**Keywords:** high-spin iron(II) porphyrin, X-ray molecular structure, UV–visible spectroscopy, IR spectroscopy, DFT calculation and MEP, NCI-RDG analyses

## Abstract

An Fe(II)-chlorido five-coordinate picket fence porphyrin complex with the formula [K (crypt-222)][Fe^II^(TpivPP)Cl]·C_6_H_5_Cl (**I**) (where TpivPP is the picket fence porphyrin and crypt-222 is the cryptand-222) has been synthesized and characterized. Cryptand-222 was used to solubilize potassium chloride . UV/Vis and IR spectroscopic data studies have also been performed. The X-ray structural analysis indicates that the Fe(II) cation is a high-spin (S = 2) porphyrin and has the ${\left(d_{xy}\right)}^{2}{\left(d_{xz}\right)}^{1}{\left(d_{yz}\right)}^{1}{\left(d_{z^{2}}\right)}^{1}{\left(d_{x^{2}-y^{2}}\right)}^{1}$ ground-state electronic configuration. The average equatorial iron-pyrrole N bond length (Fe^__^N_p_ = 2.1091(2) Å) and the distance between the iron and the 24-atom mean plane of the porphyrin ring (Fe-P_C_ = 0.57 Å) are similar to those of the reported five-coordinated Fe(II) high-spin (S =2) metalloporphyrins. Theoretical calculations on complex **I** were carried out, including (i) the optimized molecular structure using the DFT/B3LYP-D3/LanL2DZ level of theory, (ii), frontier molecular orbital (FMO) calculations, (iii) molecular electronic potential analysis (MEP), and (vi) the ELF and LOL analyses. These latter theoretical studies indicate the strong hydrogen bond linking the oxygen atom of the pivaloyl groups of the TpivPP porphyrinate and some carbon atoms of the cryptand-222.

## 1 Introduction

Iron porphyrins are commonly employed as synthetic model systems. These derivatives have primarily served as models for various hemoproteins, including myoglobin, hemoglobin, cytochrome c, and cytochromes P450 (Sch et al., 1973; La Mar and Walker, 1972; Collman et al., 1973; Hoffman et al., 1980). Since the early 1980s, metalloporphyrins, particularly iron(III), have been used as catalysts in the oxidation of several organic compounds (Groves et al., 1979). Over the last three decades, the application of porphyrin compounds has expanded across multiple fields, including chemistry, biology, physics, electronics, pharmacy, and medicine. Today, porphyrins and metalloporphyrins are used in a wide array of technical applications, such as catalysts (Kobayashi et al., 1992), solar cells (Chen), sensors (Gupta et al., 2003), supramolecular chemistry (Drain et al. 2009), photosensitizers for photodynamic therapy (PDT) (O’Connor et al., 2009), photocatalysts (Girichev et al., 2000), semiconductors (Nevin and Chamberlain, 1991), and nonlinear optics (Norwood and Sounik, 1992).

Porphyrins are molecules present in nature, but they can be synthesized. They have many applications because of their characteristic physical and chemical properties. We noticed that the majority of reported low-spin iron(II) metalloporphyrins (S = 0) are either pentacoordinated or hexacoordinated (Shi et al., 2021; Paolesse et al., 2017; Lu and Zhang, 2011; Pereira et al., 2016).

The synthesis of protected porphyrins and especially the picket fence porphyrin *meso*-tetrakis(4α-*o*-(pivalamidophenyl)porphyrin) is used to prevent the formation of μ-oxo iron(III) complexes and to stabilize anionic ligands (Hu et al., 2006).

The electronic ground states of iron(II) metalloporphyrins have been extensively studied, and the usefulness of electron spectroscopy as a spectroscopic probe for the electronic structure of Fe(II) species has been demonstrated (Hu et al., 2006). The study of high-spin iron(II) porphyrinates shows two distinct types of electronic configurations (Hu et al., 2006; Hu et al., 2005). The first is the (d_*xy*_)^2^ (d_*xz*_)^1^ (d_*yz*_)^1^ (d_*z*_^2^)^1^ (d_*x*_^2^_-*y*_^2^)^1^ electronic configuration, which is usually presented by pentacoordinated Fe(II) porphyrin complexes with anionic axial ligands. The second is the (d_*xz*_)^2^ (d_*yz*_)^1^ (d_*xy*_)^1^ (d_*z*_^2^)^1^ (d_*x*_^2^_-*y*_^2^)^1^ ground-state electronic configuration, corresponding to the pentacoordinated Fe(II) porphyrin complexes with neutral axial ligands.

For the first type, the average equatorial distance between the iron(II) center ion and the four nitrogens of the porphyrin core (Fe–Np) is approximately 2.11 Å, and the displacement of the axial ligand above the 24-atom mean plane of the porphyrin core (Fe–P_C_) is approximately 0.52 Å (Hu et al., 2006).

Ferrous porphyrin complexes that exhibit the second type of electronic configuration present shorter Fe–Np bond lengths than those of the first type of approximately 2.07 Å, along with shorter Fe–P_C_ distances compared to those of the first class of Fe(II) high-spin metalloporphyrins with values of approximately 0.35 Å (Hu et al., 2006).

We, here, describe the synthesis, UV/Vis and IR spectroscopic characterization, and the single-crystal X-ray molecular structure of complex **I**. It is interesting to notice that chlorido Fe(II) porphyrin complexes are well-described in the literature (Yu et al., 2015).

DFT simulations were used to conduct an extensive theoretical investigation of the chlorido ferrous porphyrin complex. Frontier molecular orbitals (FMOs), key stability parameters, molecular electrostatic potential (MEP), and NBO analysis were all computed in this study. By validating the experimental results and providing insights into the opto-electronic properties of complex **I**, this incorporated strategy offers a greater comprehension of its electronic structure and reactivity. The NCI and RDG indicate the presence of several electrostatic interaction complexes between groups, which may contribute to the stability of our compound within the crystal lattice.

## 2 Experimental section

### 2.1 General procedures

The chlorobenzene solvent was purified by washing with sulfuric acid and then distilled over P_2_O_5_, and *n*-hexane was distilled over CaH_2_. All solvents were degassed before use by three freeze/pump/thaw cycles. The cryptand-222 was recrystallized from toluene (dried by distillation over sodium/benzophenone) and stored under an argon atmosphere in the dark. All manipulations were carried out under argon using a double-manifold vacuum line, Schlenkware, and cannula techniques. The FTIR spectra were acquired with a SHIMADZU FTIR-8400 spectrometer, while absorption spectra were recorded using a SHIMADZU UV-2401 spectrometer.

### 2.2 Synthesis of [K(2,2,2-crypt)][Fe^II^(TpivPP)Cl]·C_6_H_5_Cl

The picket fence porphyrin (H_2_TpivPP) was synthesized according to the reported method (Colman et al., 1975). Hundred micrograms (0.081 mmol) [Fe^III^(TpivPP)(SO_3_CF_3_)(H_2_O)] (Gismelseed et al., 1990) and 1 mL of zinc amalgam were stirred for 1 h under argon in 10 mL of C_6_H_5_Cl. The *in situ* solution of [Fe^II^(TpivPP)] was subsequently filtered into a second mixture comprising 10 mL of chlorobenzene, 150 mg (0.39 mmol) of cryptand-222, and 200 mg of KCl (2.68 mmol) for a duration of 2 h. Crystals of the obtained species were formed through the gradual diffusion of *n*-hexane into the chlorobenzene solution.

Complex I (C88H105Cl2FeKN10O10) (1624.64) Calcd: C 65.06, H 6.27, and N 8.62; found: C 65.08, H 6.28, and N 8.63; UV-Vis [in C6H5Cl, λmax nm (logε)]: 440(5.88), 568(4.59), and 612 (4.54); IR [ $\text{solid},\bar{\nu }$ cm-1]: 3,417 [ν(NH)porphyrin], 2,958–2,812 [ν(CH)porphyrin], 1,680 [ν(C=O)porphyrin], 1,104 [ν(CH2-O-CH2)2,2,2-crypt], and 987 [δ(CCH)porphyrin].

### 2.3 X-ray crystallography

The diffusion of *n*-hexane through a solution of chlorobenzene produced good-quality crystals of **I**. A dark-blue crystal with the dimensions 0.48 × 0.42 × 0.23 mm^3^ was utilized for the data collection. The crystalline sample was positioned in inert oil, mounted on a glass pin, and moved to the goniometer of the diffractometer. The data collection was performed at 233 K using a Bruker APEXII CCD diffractometer, employing Mo Kα radiation with a graphite monochromator (*λ* = 0.7107 Å). The unit cell dimensions were refined based on the complete data set. The integration and scaling processes produced a data set that was adjusted for Lorentz and polarization effects through the use of DENZO/SCALEPACK (Otwinowski and Minor, 1997).

The trial structure was obtained by direct methods using SIR-2004-1.0 (Burla et al., 2005), which revealed the position of the iron and potassium atoms, most atoms of the porphyrinato ligand, and the cryptand-222. The asymmetric unit contains one molecule of [Fe^II^(TpivPP)Cl]^-^, the counterion [K(crypt-222)]^+^, and one molecule of chlorobenzene. The final structural refinement was made with *F* ^2^ data with the program shelxl-97 (Scheldrick, 2015).

Using the assumed geometry, atoms of hydrogen were positioned with C–H (aromatic = 0.95 Å and methyl = 0.98 Å). For the H atoms, the displacement parameters were set by the command to 1.2 (1.5 for methyl) times the isotropic equivalent for the bonded carbon and nitrogen atoms.

The relevant crystallographic outcomes for our complex are displayed in Table 1. Distances (Å) and angles (°) of the iron coordination polyhedron and the cation complex are given in Table 2.

**TABLE 1:** Crystal data and structural refinement for [K(crypt-222)][Fe^II^(TpivPP)Cl]·C_6_H_5_Cl.

Empirical formula	C_88_H_101_Cl_2_FeKN_10_O_10_
Formula weight (M)	1,624.64
Crystal system	Monoclinic
Space group	*P2*_*1*_/*n*
*Lattice constants*	
*a* (Å)	17.8180 (6)
*b* (Å)	21.3889 (7)
*c* (Å)	22.5728 (9)
*α* (°)	90.0
*β* (°)	100.710 (3)
*γ* (°)	90.0
Volume, *V* (Å^3^)	8,452.81 (5)
*Z*	4
*D*_calc_ (g/cm^3^)	1.277
Absorption coefficient, *μ* (mm^-1^)	0.355
*F* (000)	3432
Crystal size (mm^3^)	0.48 × 0.42 × 0.23
*T* (K)	150 (2)
*θ* range for data collection	2.64–25.99
Limiting indices	−20 ≤ *h* ≤ 21, −26 ≤ *k* ≤ 26, −27 ≤ *l* ≤ 27
Unique reflections (*R*_*σ*_)	9,361 (0.080)
Data/restraints/parameters	16,602/19/1,021
Goodness-of-fit on *F* ^2^	1.043
Final *R* indices [*I* > 2*σ*(*I*)]	*R*_1_ ^a^ = 0.0553, *wR*_2_ ^b^ = 0.1374
*R* indices (all data)	*R*_1_ = 0.1165, *wR*_2_ = 0.1585
Largest difference in peak and hole (e Å^-3^)	1.025 and −0.819
CCDC	2367774

^a^ *R*_1_ = ∑||*F*_o_| − |*F*_c_||/∑|*F*_o_|.

^b^ ${wR}_{2}={\left[\sum w{\left(F_{o}^{2}-F_{c}^{2}\right)}^{2}/\sum w{\left(F_{o}^{2}\right)}^{2}\right]}^{1/2}$ , and $w=1/\left[\sigma^{2}\left(F_{o}^{2}\right)+{\left(0.0797P\right)}^{2}+0.00P\right]$ , where $P=\left(F_{o}^{2}+{2F}_{c}^{2}\right)/3$ .

**TABLE 2:** Distances (Å) and angles (°) of the iron and the potassium coordination polyhedron and of complex **I**.

*Iron coordination polyhedron*					
Fe-N1	2.089 (2)	N1-Fe-N2	86.62 (10)	N1-Fe-Cl1	103.02 (8)
Fe-N2	2.094 (3)	N1-Fe-N3	150.79 (11)	N2-Fe-Cl1	107.29 (8)
Fe-N3	2.100 (3)	N1-Fe-N4	86.97 (10)	N3-Fe-Cl1	106.18 (8)
Fe-N4	2.080 (2)	N2-Fe-N4	152.05 (11)	N4-Fe-Cl1	100.65 (8)
Fe-Cl	2.2877 (11)	N2-Fe-N3	85.47 (10)		
*Potassium-cryptand-222*					
K-N9	3.013 (3)	K-O8	2.781 (3)	N9-K-O8	120.99 (9)
K-N10	3.044 (3)	K-O9	2.784 (3)	N10-K-O5	119.47 (8)
K-O5	2.827 (2	K-O10	2.815 (2)	N9-K-O5	60.18 (8)
K-O6	2.818 (2)	N9-K-N10	179.65 (9)	N9-K-O6	119.60 (8)
K-O7	2.822 (2)	N9-K-O7	61.11 (8)	N9-K-O9	59.91 (9)

## 3 Results and discussion

### 3.1 Synthesis of complex I

The free picket fence porphyrin with the symbol H_2_TpivPP and the triflato iron(III) porphyrinic complex were synthesized according to the reported methods (Colman et al., 1975; Gismelseed et al., 1990). The reaction schemes leading to the preparation of the *meso*-arylporphyrin and the two complexes chlorido and triflato of iron(III) are given in Supplementary Schemes 1–3. This type of protected porphyrin is chosen because it is known to stabilize iron(II) metalloporphyrins with anionic axial ligands (Dhifet et al., 2010a; Nasri et al., 2000a; Hachem et al., 2009). The use of unprotected porphyrins, such as *meso*-tetratolylporphyrin (H_2_TTP), leads to the formation of the μ-oxo complex [Fe^III^(TTP)]_2_O (Li et al., 1999).

**SCHEME 1:**
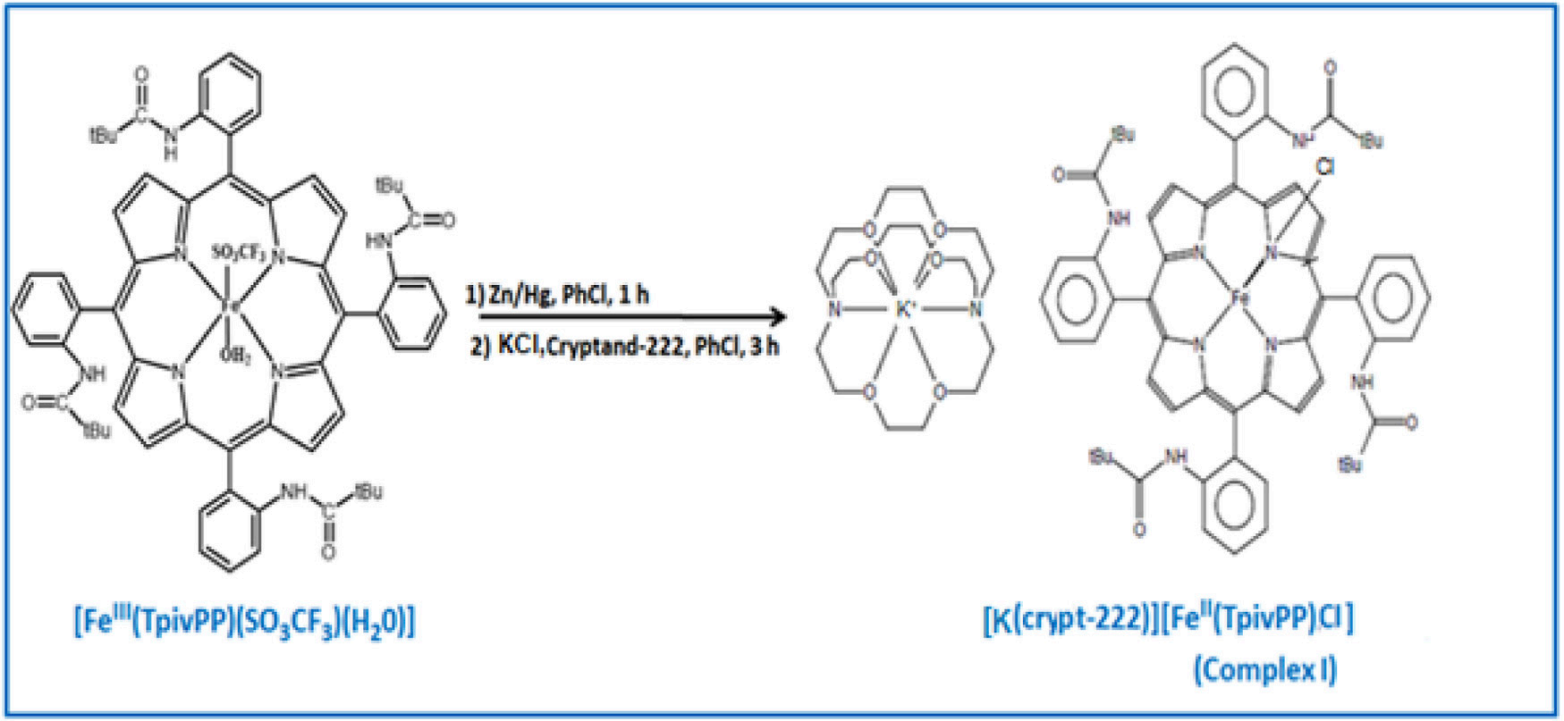
Synthesis of compound **I**.

To synthesize our compound, [Fe^III^(TpivPP)(SO_3_CF_3_)(H_2_O)] is allowed to react with zinc amalgam in chlorobenzene, which is then filtered into a chlorobenzene solution containing a mixture of cryptand-222 and potassium chloride. After 3 h of reaction at room temperature and under argon, [K(crypt-222)][Fe^II^(TpivPP)Cl] (**I**) is formed (Scheme 1).

### 3.2 Spectroscopic characterizations

**FIGURE 1:**
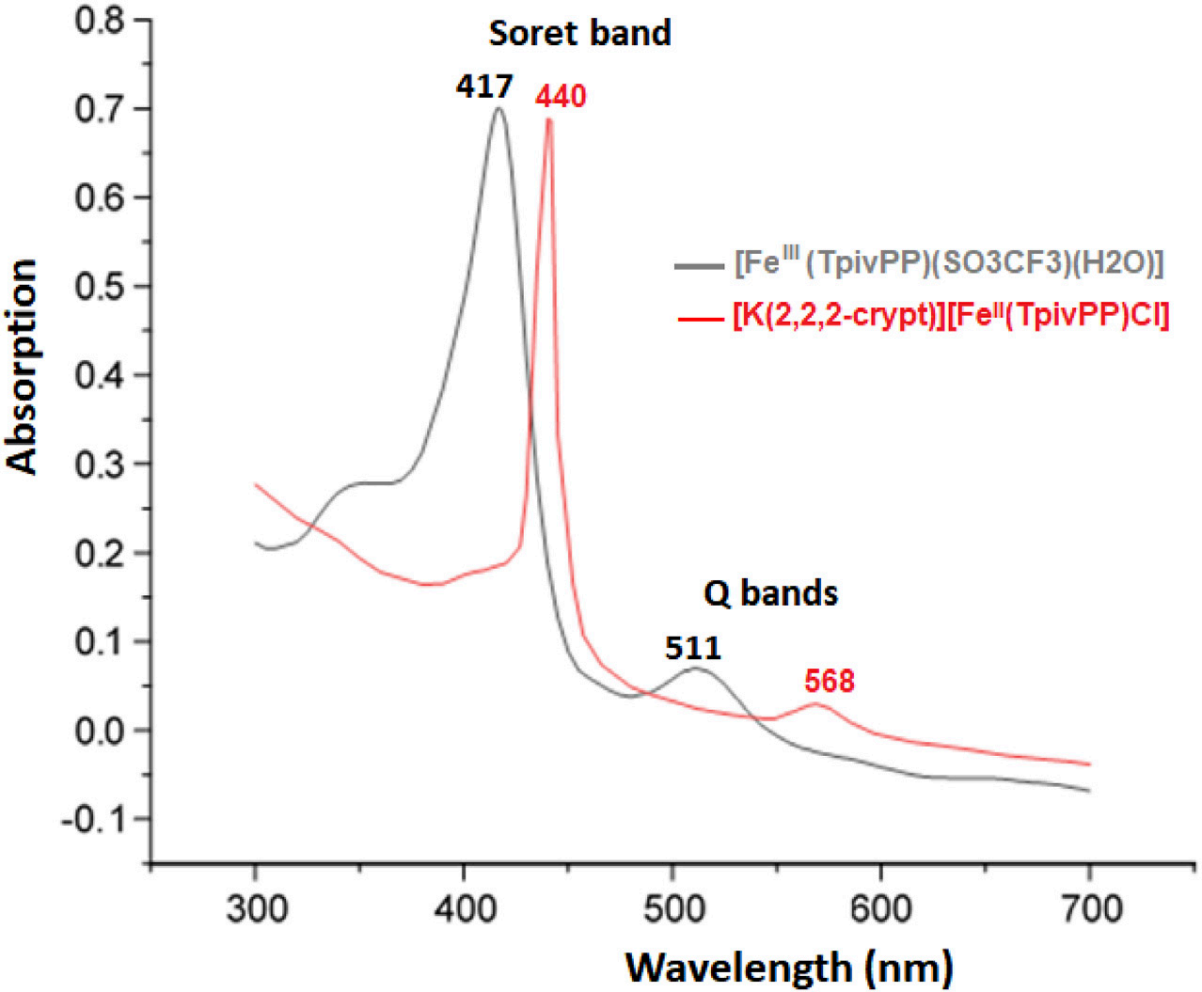
UV/Vis absorption spectra of the [Fe^III^(TpivPP)(SO_3_CF_3_)(H_2_O)] starting material and the [K(crypt-222)][Fe^II^(TpivPP)Cl]·C_6_H_5_Cl (**I**) recorded in C_6_H_5_Cl with a concentration of C ∼10^−6^ mol.L-1.

Figure 1 depicts the spectra of complex **I** and the [Fe^III^(TpivPP)(SO_3_CF_3_)(H_2_O)] starting material. The position of the Soret band at 440 nm in chlorobenzene clearly shows the deviation of this band toward the red line. The fact that this type of compound exhibits Soret bands that are strongly shifted toward the red line is explained by the charge effect: the negative charges of the ligand and the complex ion. As shown in Table 3, the Soret band of our compound at 440 nm is in the range [437–455] nm, which characterizes iron(II) five-coordinate *meso*-arylporphyrin complexes with anionic axial ligands. It can be concluded that the synthesized compound (**I**) is very similar to the reported low-spin (S = 0) and high-spin (S = 2) pentacoordinated iron(II) porphyrin complexes (Table 3). According to the UV/Vis results, complex **I** is an iron(II) five-coordinated porphyrin complex, but the spin state of iron(II) is not confirmed; this can be determined using other characterization techniques.

**TABLE 3:** Electronic spectra data for complex **I** and a selection of picket fence Fe(II) five-coordinated ion complexes.

Complex	*λ*_max_ (nm)			Spin *S*	Reference
	Soret region	α, β region			
[Fe^II^(TpivPP)(NO_2_)]^-^	444	567	608	0	Nasri et al. (1991)
[Fe^II^(TpivPP)(CN)]^-^	455	565	601	0	(Ben Abbes and Nasri)
[Fe^II^(TpivPP)(NCO)]^-^	437	568	610	2	Dhifet et al. (2010a)
[Fe^II^(TpivPP)(N_3_)]^-^	443	572	612	2	Hachem et al. (2009)
[Fe^II^(TpivPP)(OAc)]^-^	448	572	611	2	Nasri et al. (1987)
[Fe^II^(TpivPP)(OMe)]^-^	456	580	622	2	Nasri et al. (1987)
[Fe^II^(TpivPP)(NO_3_)]^-^	438	564	604	2	Nasri et al. (2006)
[Fe^II^(TpivPP)Cl]^-^	440	568	612	2	This work

Solvent: chlorobenzene.

The optical gap (Eg-opt) value of complex **I** was calculated using the following formula and the tangent method (see Supplementary Figure 1):

$$E_{g-opt}=\frac{1240}{\lambda_{Lim}}$$

Our new ferrous complex is a semiconductor since its experimental E_g-opt_ is equal to 1.90 eV.

**FIGURE 2:**
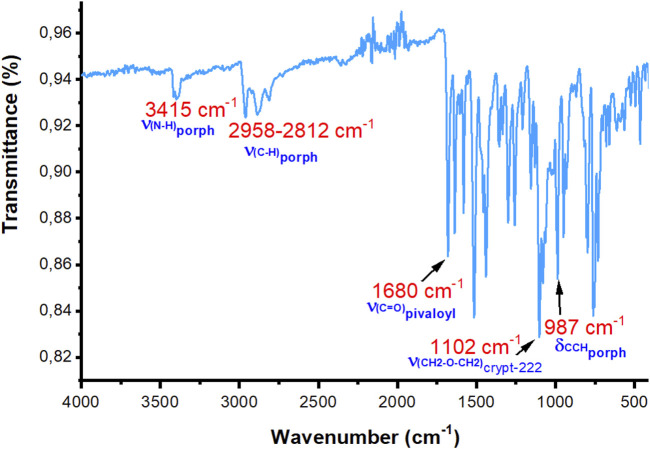
Experimental IR spectrum of compound **I** (solid state, without KBr).

The IR spectra of the H_2_TpivPP free base porphyrin and the [Fe^III^(TpivPP)(SO_3_CF_3_)] starting material are depicted in Supplementary Figures S2, S3. The experimental IR spectrum of complex **I** is shown in Figure 2. The values of the sensitive bands of some porphyrin complexes of iron(II) and iron(III) with the porphyrin TpivPP are given in Table 4.

**TABLE 4:** Values of the sensitive bands of complex **I** and some picket fence porphyrin iron(II) and iron(III) porphyrinic complexes.

Complexes	ν(NH)_porph_	ν(CO)_porph_	δ(CCH)_porph_	Reference
Iron (II) pentacoordinated				
[Fe^II^(TpivPP)N_3_]^-^	3,408	1,985	985	Hachem et al. (2009)
[Fe^II^(TpivPP)(CN)]^-^	3,419	1,683	988	(Ben Abbes and Nasri)
[Fe^II^(TpivPP)(OMe)]^-^	3,418	1,685	985	Nasri et al. (1987)
[Fe^II^(TpivPP)(OAc)]^-^	3,414	1,682	989	Nasri et al. (1987)
[Fe^II^(TpivPP)(NO_3_)]^-^	3,418	-	984	Nasri et al. (2006)
[Fe^II^(TpivPP)Cl]^-^	3,415	1,680	987	This work
Iron(III) pentacoordinated				
[Fe^III^(TpivPP)(OAc)]	-	1,670	996	Nasri (1987)
[Fe^III^(TpivPP)(NCS)]	-	1,680	998	Nasri and Debbabi (1998)
[Fe^III^(TpivPP)(NCO)]	3,430	1,672	998	Belkhiria et al. (2005)
Iron(II) hexacoordinated				
[Fe^II^(TpivPP)(NO_2_)(CO)]^-^	3,421	-	996	Nasri et al. (2004)
[Fe^II^(TpivPP)(N_3_)(CO)]^-^	3,405	-	992	Safo et al. (1990)

H_2_TpivPP exhibits the characteristic IR spectrum of a *meso*-arylporphyrin, with the ν(NH) stretching frequency observed at 3,430 cm-1 and the ν(CH) stretching frequency falling in the [2,960–2,869] cm^-1^. The δ(CCH) bending frequency value is 967 cm^-1^. The metalation of the H_2_TpivPP free base porphyrin with iron(II) chloride dihydrate FeCl_2_·2H_2_O leads to [Fe^III^(TpivPP)Cl], which reacts with the silver triflate AgSO_3_CF_3_ to give [Fe^III^(TpivPP)(SO_3_CF_3_)(H_2_O)], the starting material. Consequently, the band attributed to the ν(NH) stretching frequency of the inner pyrrolic hydrogen atoms disappears. The bending frequency δ(CCH) of the H_2_TpivPP porphyrin shifts toward the high fields from 967 cm^-1^ to 998 cm^-1^ for the iron(III) triflate starting materials and to 987 cm^-1^ for our ferrous porphyrinic complex.

[K(crypt-222)][Fe^II^(TpivPP)Cl]·C_6_H_5_Cl (**I**) presents a strong band in the IR spectrum at 1,102 cm^-1^, which is attributed to the CH_2_-O-CH_2_ stretching frequency of the cryptand-222. This confirms the presence of the counterion [K(crypt-222)]^+^. The presence of the characteristic ν(-CH_2_-O-CH_2_-) vibration frequency of the [K(crypt-222)]+ counterion and the δ(CCH)_porph_ bending vibration of the porphyrin at 987 cm^-1^ confirmed the formation of the five-coordinate iron(II) porphyrin species with ionic axial ligands (Table 4).

### 3.3 Crystal structure of complex I

**FIGURE 3:**
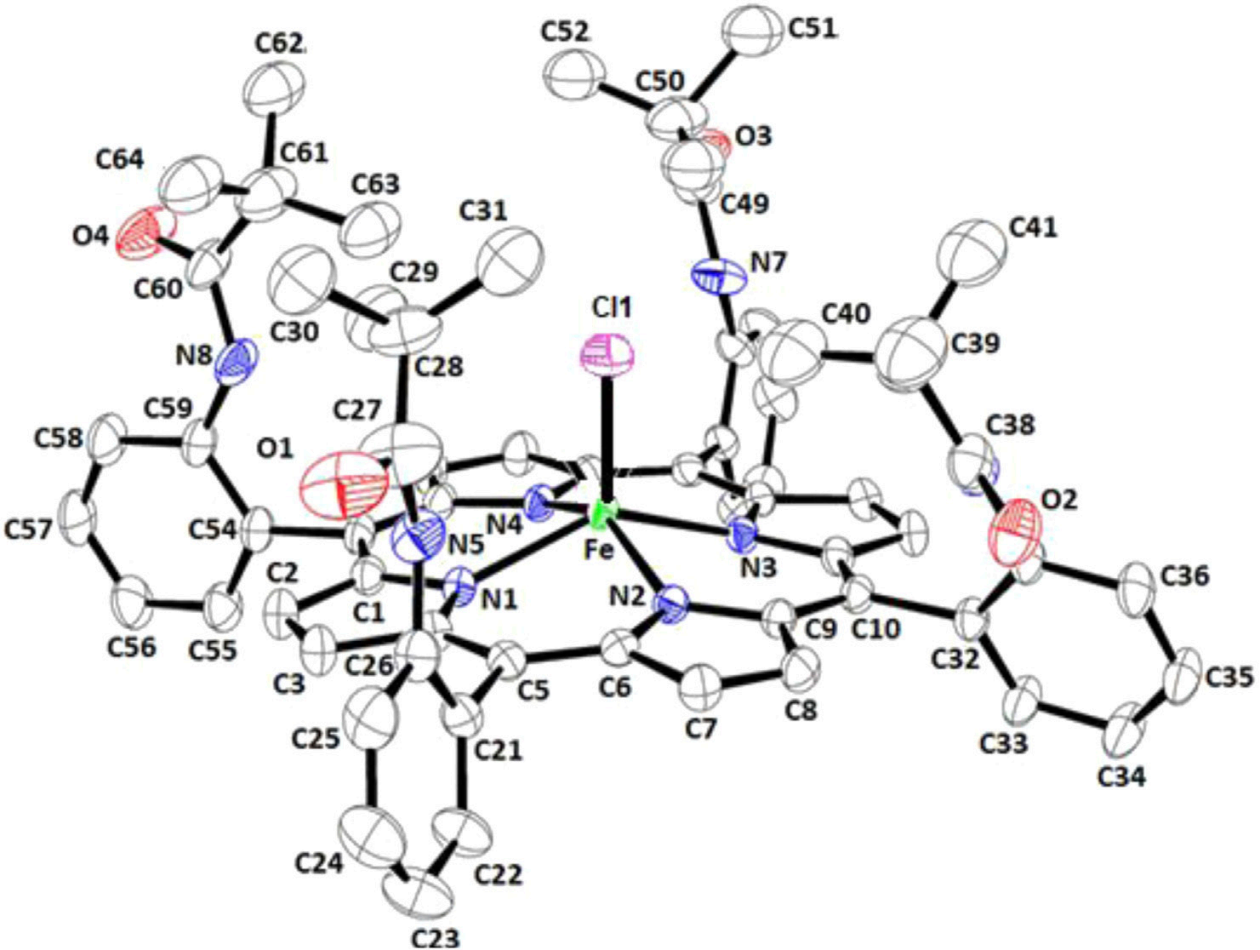
ORTEP view of the [Fe^II^(TpivPP)Cl]^−^ ion complex, where thermal ellipsoids are drawn at the 30% probability level. Hydrogen atoms have been omitted for clarity.

Figure 3 depicts an ORTEP diagram of the [Fe^II^(TpivPP)Cl]^-^ ion complex (Farrugia, 1997). The Fe(II) center metal is coordinated to the four nitrogen atoms of the TpivPP porphyrinato and the chlorido axial ligand from the pocket side of the TpivPP porphyrin.

**FIGURE 4:**
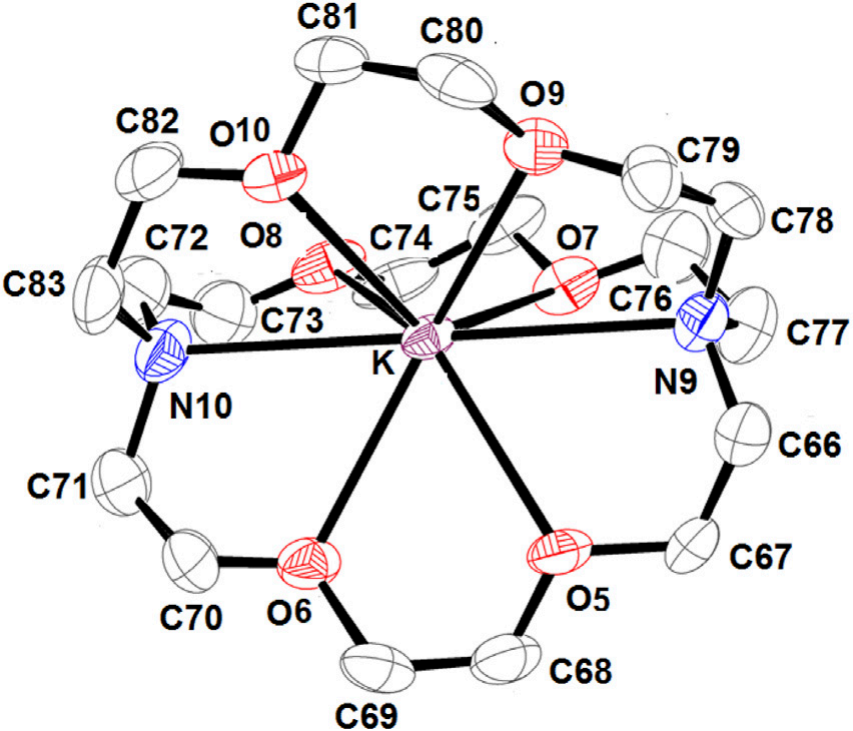
ORTEP view of [K(crypt-222)]^+^ counterion, where the thermal ellipsoids are drawn at the 30% probability level. Hydrogen atoms have been omitted for clarity.

Supplementary Figure 4 and Figure 4 illustrate the ORTEP diagrams of the [K (crypt)][Fe^II^(TpivPP)Cl]·C_6_H_5_Cl complex and the [K (crypt-222)]^+^ counterion, respectively.

The potassium atom is eight-coordinated, where it is coordinated to two nitrogen atoms and six oxygen atoms of the cryptand-222. The average K–O (crypt-222) distance is 2.807 (3) Å, and the average K–N (crypt-222) bond length is 3.028 (3) Å.

**FIGURE 5:**
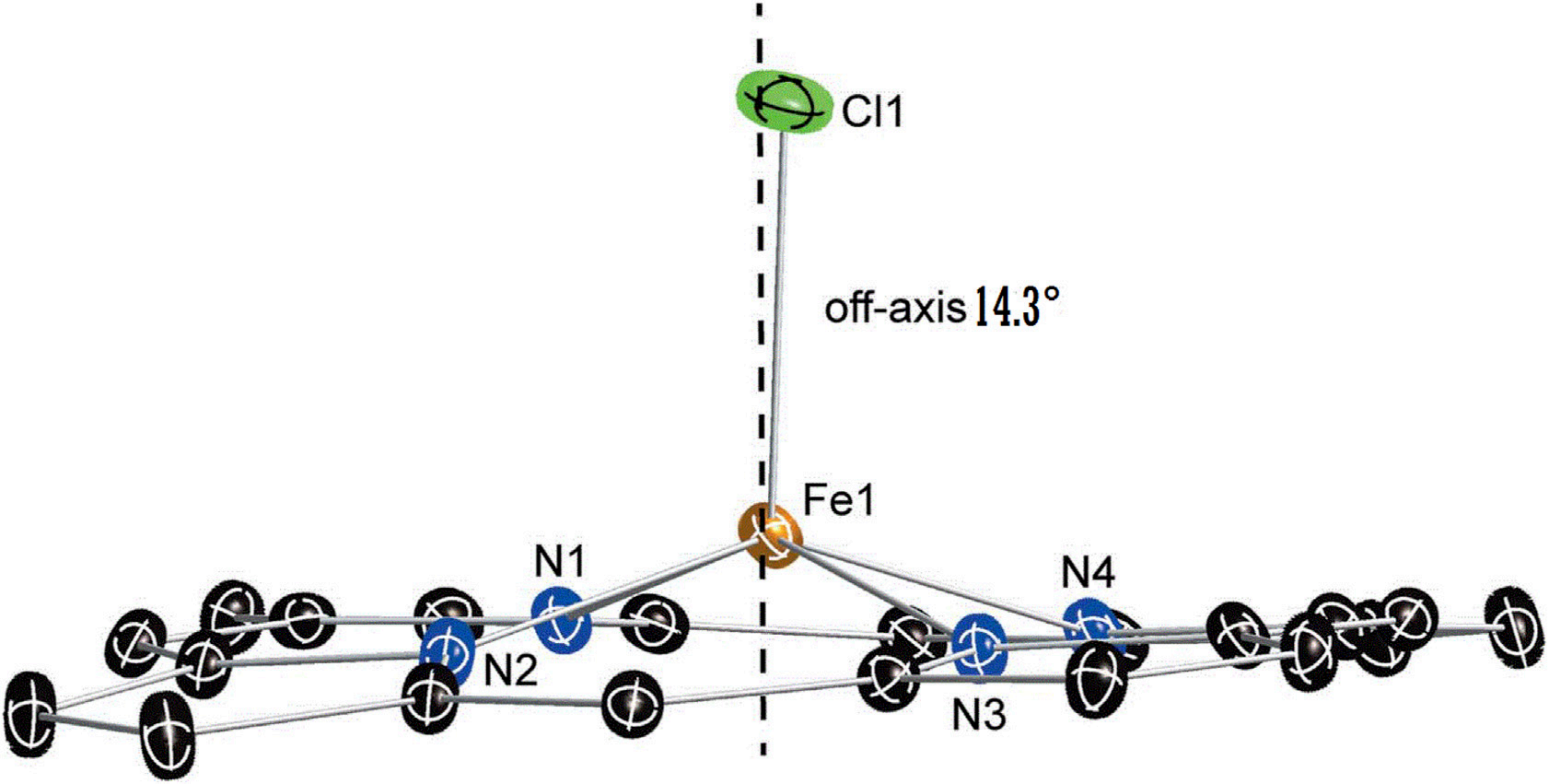
Diagram illustrating the off-axis tilt of the axial ligand, the near-planar conformation, and the core dome of the [Fe^II^(TpivPP)Cl]^-^ ion complex. The dashed line denotes the position of the normal to the porphyrin plane.

Figure 5 shows that for our chlorido iron(II) porphyrin complex, the iron atom is bonded to the four pyrrole nitrogen atoms (Np) of the porphyrin core and to the Cl^−^ axial ligand. The Fe–Cl bond is tilted slightly to the porphyrin plane by 14.3°.

This Fe–Cl vector tilt could be explained by the interactions between the Cl^−^ ion, which is coordinated to the iron(II) inside the pocket of the porphyrin, and the closest hydrogen atoms of the *t*-butyl groups of the four pivaloyl moieties of the picket fence porphyrin. Indeed, the shortest Cl… H interactions present a quite short distance between 3.39 and 2.91 Å (Supplementary Figure 5). Table 5 summarizes several distance values concerning the coordination sphere of a selection of iron(III) and iron(II) metalloporphyrins. Notably, as shown in Table 5 the Fe–Cl distance is slightly longer for an iron(II) metalloporphyrin than that of an iron(III) porphyrin complex, which is due to the smaller size of Fe^3+^ compared to Fe^2+^.

**TABLE 5:** Porphyrinato core parameters (Å) for selected Fe(II) and Fe(III) *meso*-porphyrin complexes.

Complex	Fe-N_p_ ^a^	Fe-X_L_ ^b^	Fe-P_C_ ^c^	Fe-P_N_ ^d^	Reference
*Iron(II) high-spin (S = 2) metalloporphyrins*					
[Fe^II^(TpivPP)Cl]^-^	2.1091 (2)	2.2877 (11)	0.57	0.51	This work
[Fe^II^(TpivPP)(N_3_)]^-^	2.094 (3)	2.078 (2)	0.52	0.46	Hachem et al. (2009)
[Fe^II^(TpivPP)(OAc)]^-^	2.107 (2)	2.034 (3)	0.64	0.55	Nasri et al. (1987)
[Fe^II^(TpivPP)(NO_3_)]^-^	2.070 (2)	2.069 (4)	0.49	0.42	Nasri et al. (2006)
[Fe^II^(TpivPP)(NCO)]^-^	2.120 (2)	2.005 (3)	0.68	0.59	Dhifet et al. (2010b)
[Fe^II^(TpivPP)(NCS)]^-^	2.104 (2)	2.042 (2)	0.59	0.50	Dhifet et al. (2010b)
*Iron(II) low-spin (S = 0) metalloporphyrins*					
[Fe^II^(TpivPP)(NO_2_)]^-^	1.970 (4)	1.849 (6)	0.17	0.13	Nasri et al. (2000b)
[FeII(TPP)(CN)_2_]^2-^	1,986 (8)	1.878 (1)	0.22	0.13	Li et al. (2008)
*Iron(III) high-spin*					
[Fe^III^(TpivPP)(NCO)]	2.069 (5)	1.970 (5)	0.54	0.49	Belkhiria et al. (2005)
[Fe^III^(TPP)Cl]	2.070 (3)	2.211 (5)	0.57	–	Schiedt and Finnegan (1989)
[Fe^III^(TpivPP)Cl]	2.108 (2)	2.207 (1)	0.42	–	Dhifet et al. (2011)
[Fe^III^(TTP)(OAc)]^e^	2.067 (3)	1.898 (4)	0.52	0.48	Oumous et al. (1984)

^a^ The average distance between iron and nitrogen in the equatorial pyrrole.

^b^ The distance from iron to the axial ligand.

^c^ The distance from iron to the average plane formed by the 24-atom core of the porphyrin.

^d^ The distance from the iron center to the average plane of the four nitrogen atoms in the pyrrole.

^e^ TTP refers to tetratolylporphyrin.

For complex **I**, the Fe–Cl distance value is 2.2877 (11) Å, which is higher than that of [Fe^III^(TpivPP)Cl], with a Fe–Cl distance of 2.207 (1) Å.

Additionally, the Fe–N_p_ distance for the [Fe^II^(TpivPP)Cl]^-^ ion is 2.092 (3) Å, which is consistent with five-coordinate high-spin iron(II) porphyrins, which typically exhibit distances ranging from 2.072 Å to 2.115 Å (Guilard et al., 1987). This observation supports the conclusion that our new synthetic compound is of high-spin (S = 2) type.

Scheidt and Reed (1981) reported that for iron(II) metalloporphyrins, there is a relationship between the spin-state of the iron(II) and the value of the average equatorial iron-pyrrole N atoms distance (Fe–Np). Thus, for high-spin (S = 2) iron(II) porphyrins with weak crystal field axial ligands such as halides and pseudo-halides, the Fe–Np bond length values are large; for example, for the [Fe^II^(TpivPP)(N_3_)]^-^ ion complex (Hachem et al., 2009), the Fe–Np distance is 2.094 (3) Å, and it is 2.120 (2) Å for the [Fe^II^(TpivPP)(NCO)]^-^ ion complex (Dhifet et al., 2010b). For low-spin (S = 0) porphyrins, the Fe–Np distance is smaller. This is the case of the iron(II) metalloporphyrins with strong crystal field axial ligands. Thus, for the nitrito-*N* derivative [Fe^II^(TpivPP)(NO_2_)]^-^ ion complex, which is a low-spin Fe(II) species, the Fe–Np distance is 1.970 (4) Å (Nasri et al., 2000b) (Table 5). For complex **I**, the Fe–Np distance is 2.1091 (2), which is an indication that our [Fe^II^(TpivPP)Cl]^-^ ion complex (**I**) is high-spin (S = 2).

**FIGURE 6:**
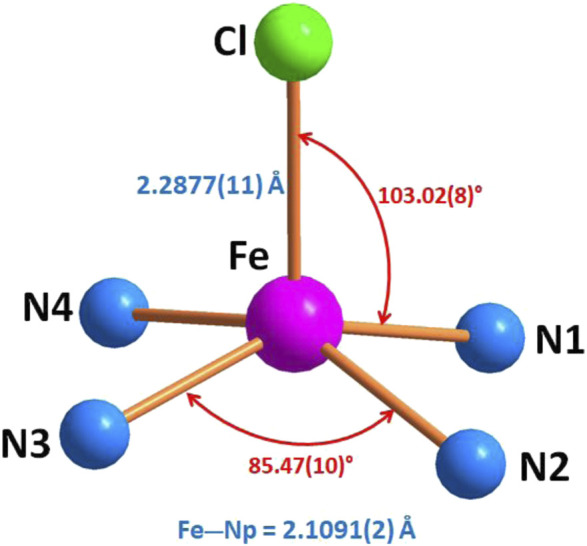
Geometric parameters (distances (Å) and angle (°)) of the iron coordination polyhedron.

The porphyrinato core undergoes significant radial expansion to accommodate the high-spin iron(II) atom. This phenomenon is further illustrated by the long Fe–Pc (where P_C_ refers to the plane made by the 24-atom core of the porphyrin) and Fe–P_N_ (where P_N_ refers to the plane made by the four nitrogens of the porphyrin ring) distances observed in [Fe^II^(Porph)(L)]^-^ complexes, where L is an anionic ligand, as detailed in Table 5. Figure 6 represents the coordination polyhedron of the chlorido iron(II) picket fence derivative (**I**). Thus, the Fe(II) cation is located in the center of the porphyrin core and defines a distorted square pyramidal environment of four nitrogen atoms of the porphyrin macrocycle and the chlorido axial ligand inside the hydrophobic cavity of the porphyrin.

**FIGURE 7:**
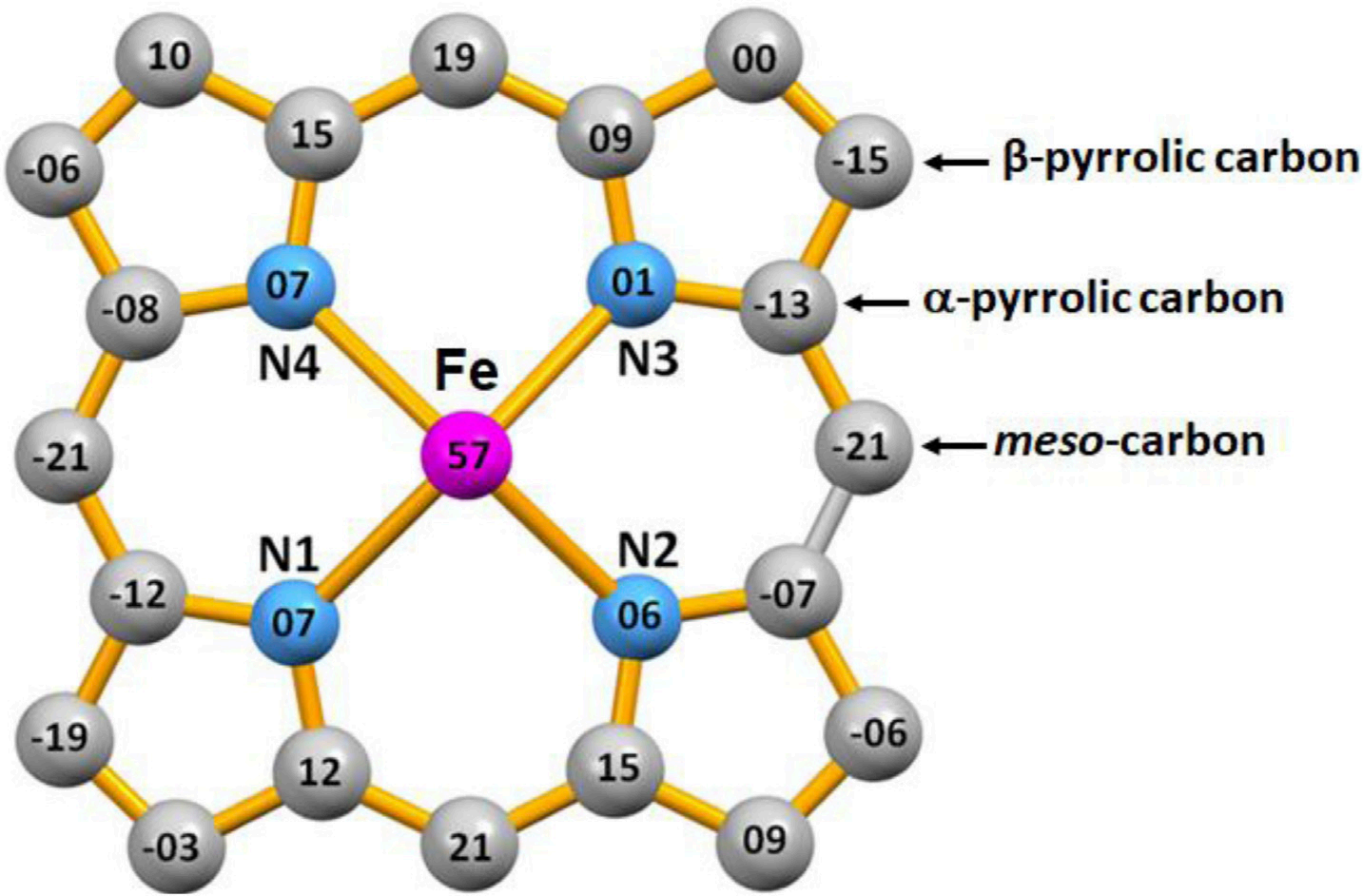
Formal diagram of the porphyrinato core of [Fe^II^(TpivPP)Cl]^-^ illustrating the displacement, in units of 10-2 Å, of each atom from the mean plane of the 24-atom porphyrin core. Positive values of displacement are toward the chloride axial ligand.

Figure 7 represents the formal diagram of the porphyrinato core of the [FeII(TpivPP)Cl]- ion complex illustrating the displacement, in units of 10–2 Å, of each atom from the mean plane of the porphyrin macrocycle. This diagram shows that the porphyrin macrocycle presents a small doming and quite significant ruffling.

**FIGURE 8:**
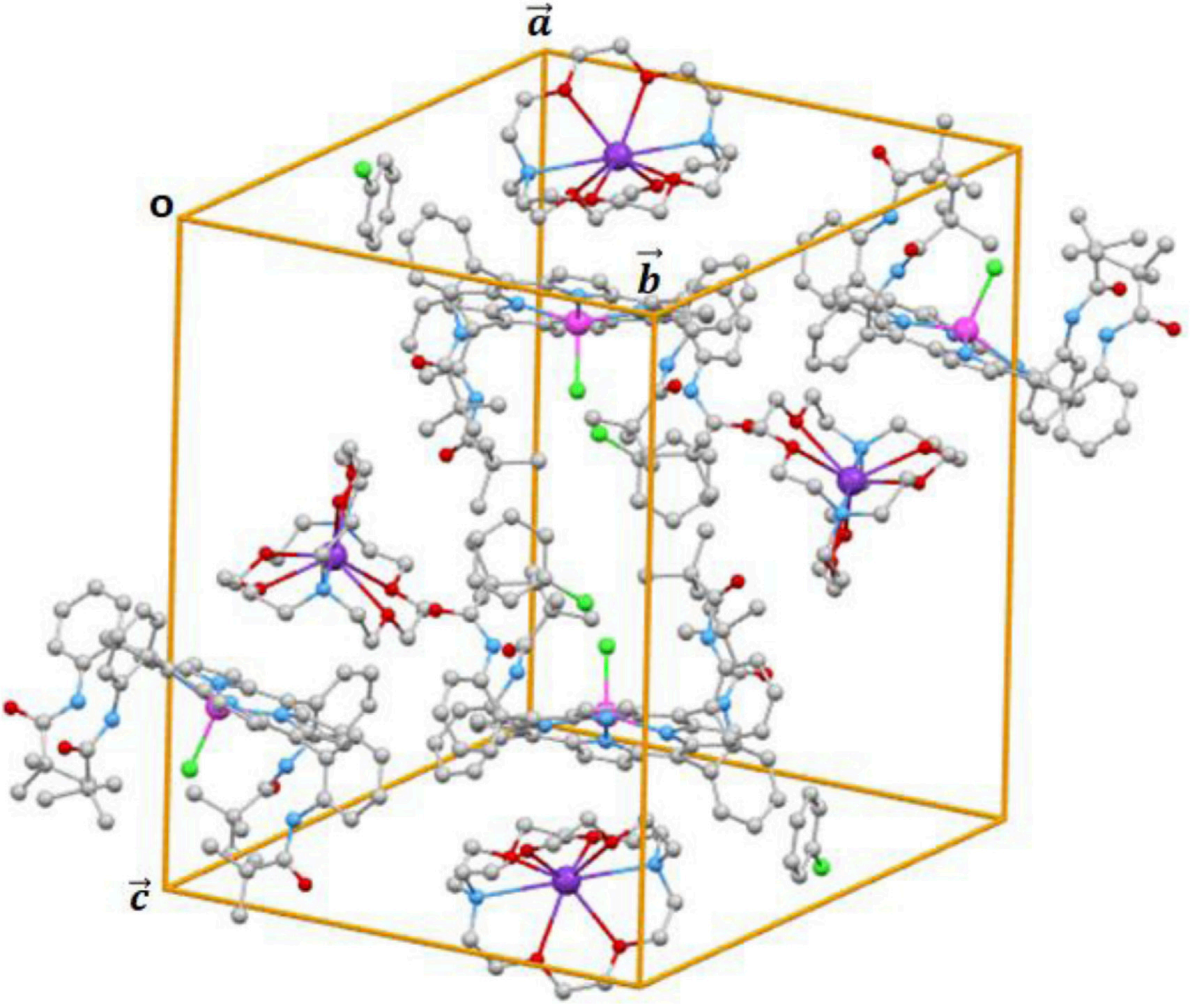
Cell content of complex **I**.

The content of the unit cell is depicted in Figure 8, which is made by four [Fe^II^(PivPP)Cl]^-^ ion complexes, four [K (crypt-222)]^+^ counterions, and four chlorobenzene solvent molecules.

The intermolecular interactions within the crystal lattice could be obtained using the PLATON program (PLATON, a multipurpose crystallographic tool). The different types of intermolecular interactions that could be detected using the PLATON program are: classic hydrogen bands such as (i) O–H…O and O–H…N interactions; (ii) non-conventional hydrogen bonds such as C–H…O and C–H…N interactions; (iii) non-conventional H bonds such as C–H…Cg, where Cg is the centroid of a pyrrole and phenyl rings for porphyrinic compounds; and (iv) π–π stacking (Cg…Cg) interactions (Dey et al., 2024; Sarkar et al., 2024).

For complex **I**, the intermolecular interactions responsible for the stability of the crystal lattices are of types C–H…O, C–H…N, and C–H…Cg (Cg is the centroid of a pyrrole or phenyl rings). The PLATON program (PLATON, a multipurpose crystallographic tool) shows that for complex **I**, the π–π stacking interactions present Cg…Cg distances superior to 4.45 Å, and this is why we did not take these interactions into account.

**FIGURE 9:**
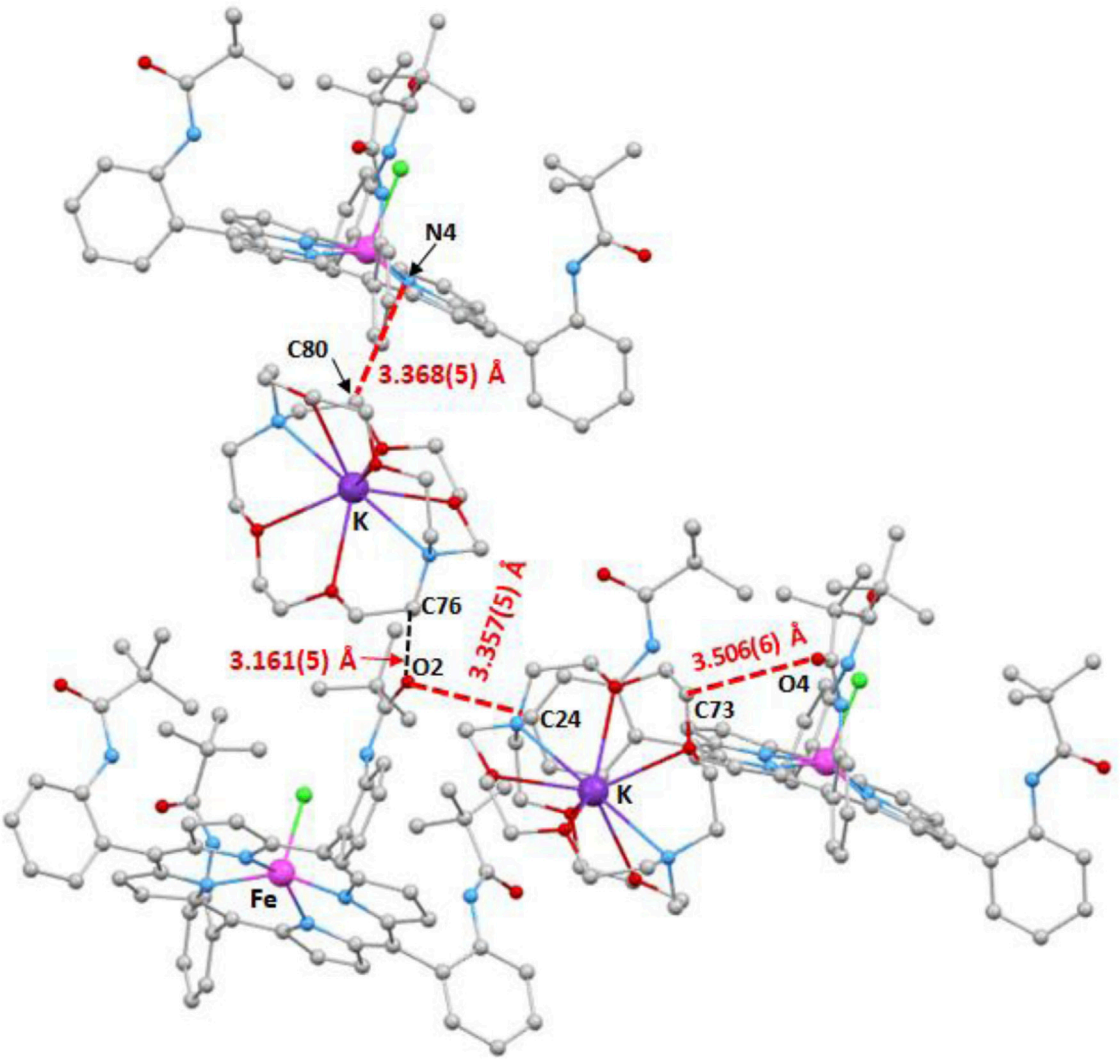
Illustration of the C–H…O and C–H…N intermolecular interactions in the crystal lattice of complex **I**.

**FIGURE 10:**
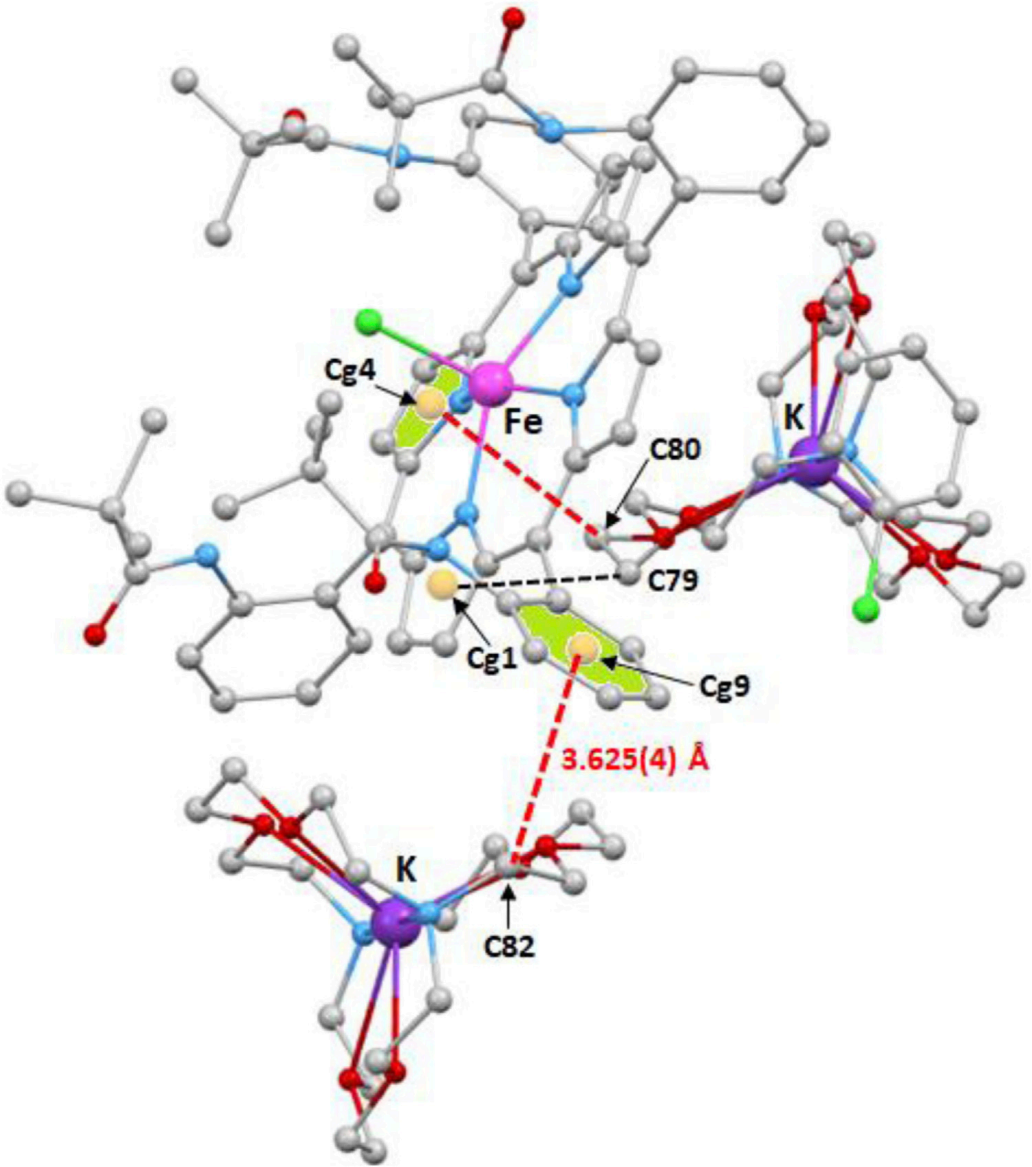
Illustration of three C–H…Cg intermolecular interactions in the crystal lattice of complex **I**.

**FIGURE 11:**
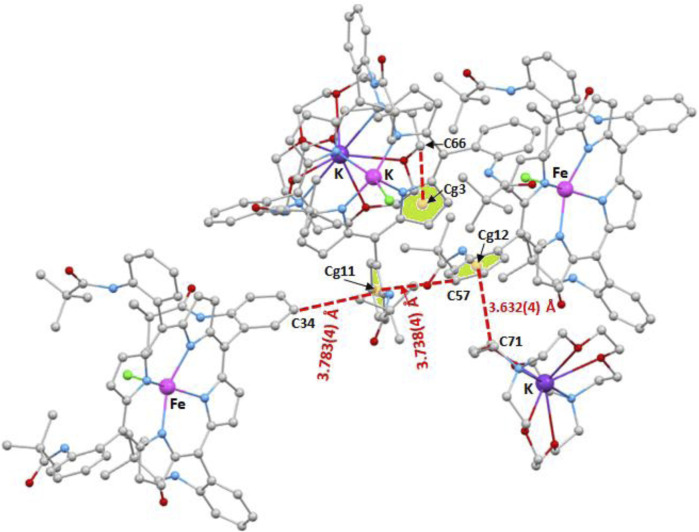
Illustration of four C–H…Cg intermolecular interactions in the crystal lattice of complex **I**.

The intermolecular interactions within the crystal lattice of complex **I** were visualized using the MERCURY program (Sarkar et al., 2024). These intermolecular contacts are depicted in Figures 9–11, while the values of these distances are given in Supplementary Table 1.

As shown in Figure 9, the O_2_ of the carbonyl of one pivaloyl group of one [Fe^II^(TpivPP)Cl]^-^ ion complex is H-bonded to the carbon C_24_ of one phenyl ring of a nearby [Fe^II^(TpivPP)Cl)]^-^ ion complex with a C_24_–H_24_…O_2_ distance of 3.357(5) Å. This O_2_ atom is also linked to the carbon C_76_ of a close [K(cryp-222)]^+^ counterion with a C_76_–H_76_B…O_2_ distance of 3.161(5) Å. The oxygen O_4_ of another carbonyl group of the TpivPP porphyrinate of one [Fe^II^(TpivPP)Cl]^-^ ion complex and the carbon C_73_ of a neighboring [K(cryp-222)]^+^ counter-ion are linked by a weak H bond with a C_73_–H_73_B…O_4_ distance of 3.506(6) Å. One [Fe^II^(TpivPP)Cl]^-^ counterion and one closely [K(crypt-222)]^+^ counterion are weakly H-linked via the carbon C_80_ of the [K(crypt-222)]^+^ ion and the nitrogen N_4_ of a pyrrole ring of the [Fe^II^(TpivPP)Cl]- ion with a C_80_–H_80_A…N_4_ distance of 3.368(5) Å.

Figures 10, 11 illustrate the C–H…Cg (Cg is the centroid of a pyrrole or a phenyl ring of the TpivPP porphyrinate) intermolecular interactions. Thus, the centroids Cg1 and Cg4 of two pyrrole rings of one [Fe^II^(TpivPP)Cl]^-^ ion complex are hydrogen bonded to the carbons C_76_ and C_80_ of a neighboring [K(crypt-222)]^+^ ion complex with a C_76_–H_76_B…Cg1 and C_80_–H_80_A distances of 3.957(4) Å and 3.387(4) Å, respectively (Figure 10). The same [Fe^II^(TpivPP)Cl]^+^ ion is linked to another [K(crypt-222)]^+^ ion via the C_82_–H_82_B…Cg9 interaction (Cg9 is the centroid of a phenyl ring, and C_82_ is a carbon atom of a cryptand-222) with a distance of 3.625(4) Å. On the other hand, the centroid Cg11 of a phenyl ring of one [Fe^II^(TpivPP)Cl]^-^ is H-bonded to the carbon C_34_ and the carbon C_57_ of two nearby [Fe^II^(TpivPP)Cl]^-^ ion complexes with C_34_–H_35_…Cg11 and C_57_–H_57_…Cg11 values of 3.783(4) Å and 3.738(4) Å, respectively. The centroid Cg12 of a phenyl ring of an [Fe^II^(TpivPP)Cl]^-^ ion complex is H-bonded to the carbon C_71_ with a C_71_–H_71_B…Cg12 distance of 3.632(4) Å. The carbon C_66_ of a [K(cryp-222)]^+^ counterion is weakly hydrogen-bonded to the centroid Cg3 of a pyrrole ring of a nearby [Fe^II^(TpivPP)Cl]^-^ ion complex with a C_66_–H_66_…Cg3 distance of 3.894(4) Å (Figure 11).

### 3.4 DFT-computational investigations of compound I

#### 3.4.1 Optimized structure

**FIGURE 12:**
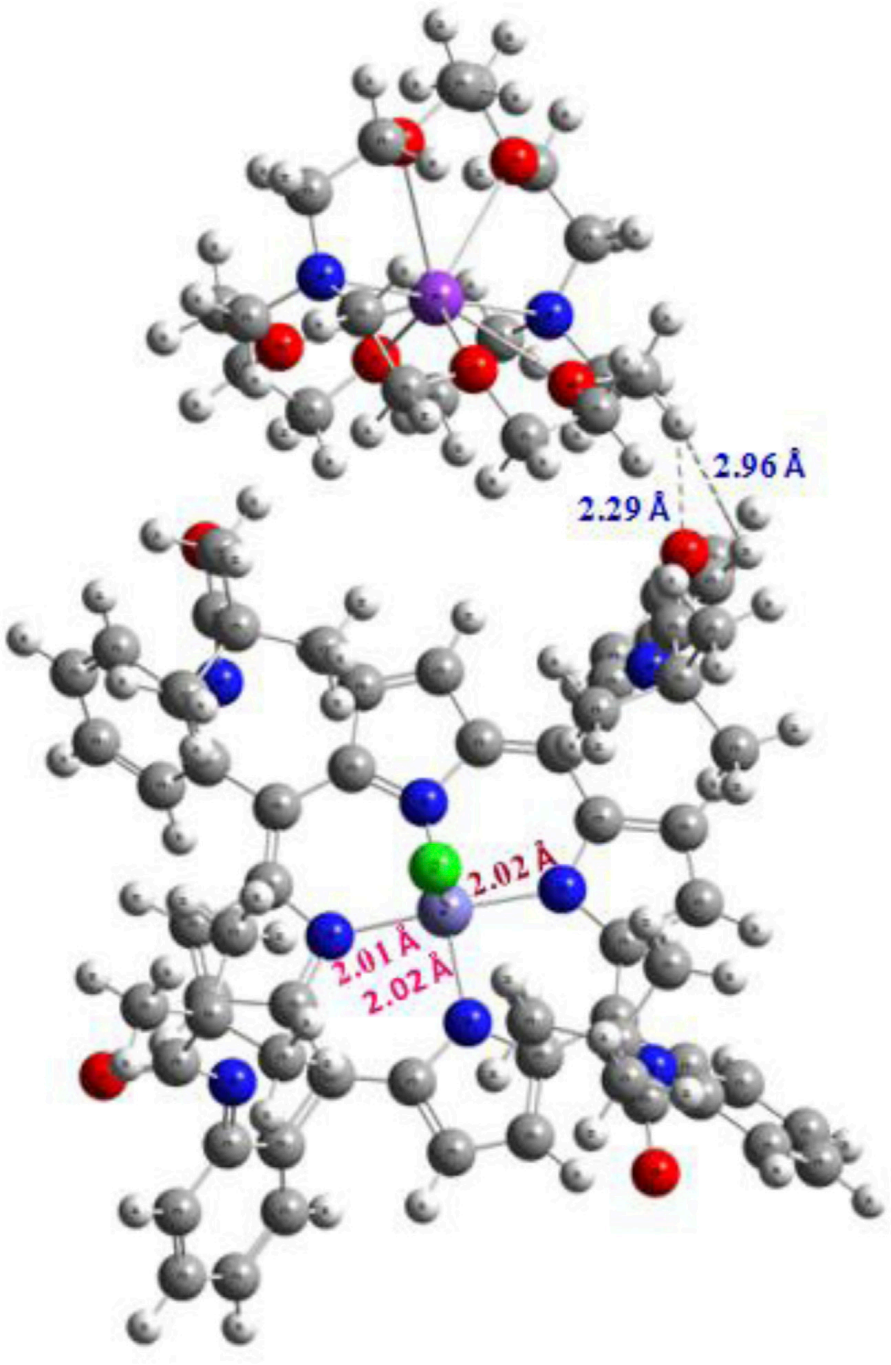
Optimized structure of the studied complex.

The CIF file generated from X-ray diffraction analysis was input into Gaussian 16 (Frisch et al., 2016) and subsequently optimized using the DFT/B3LYP/LanL2DZ level of theory (Dardouri et al., 2023; Majumdar et al., 2024a; Majumdar et al., 2024b; Mkacher et al., 2025). GaussView 6 (Roy et al., 2009) was employed as the visualization software. The optimized structure of our new compound is illustrated in Figure 12. Notably, the distance between the iron (Fe) atom distant from the N-ring is measured at 2.02 Å and 2.01 Å, while the corresponding experimental values are 2.08 Å and 2.09 Å, respectively. Furthermore, the counterion interacts with the Fe-porphyrin through H…H and H…O interactions, which are localized at distances of 2.96 Å and 2.29 Å, respectively. The experimental measurements for these interactions are approximately 3.16 Å and 2.28 Å, respectively. These findings demonstrate a good agreement between the experimental and theoretical results, indicating a well-stabilized atomic arrangement within the studied system and enhancing its potential applicability.

#### 3.4.2 HOMO/LUMO isosurface

**FIGURE 13:**
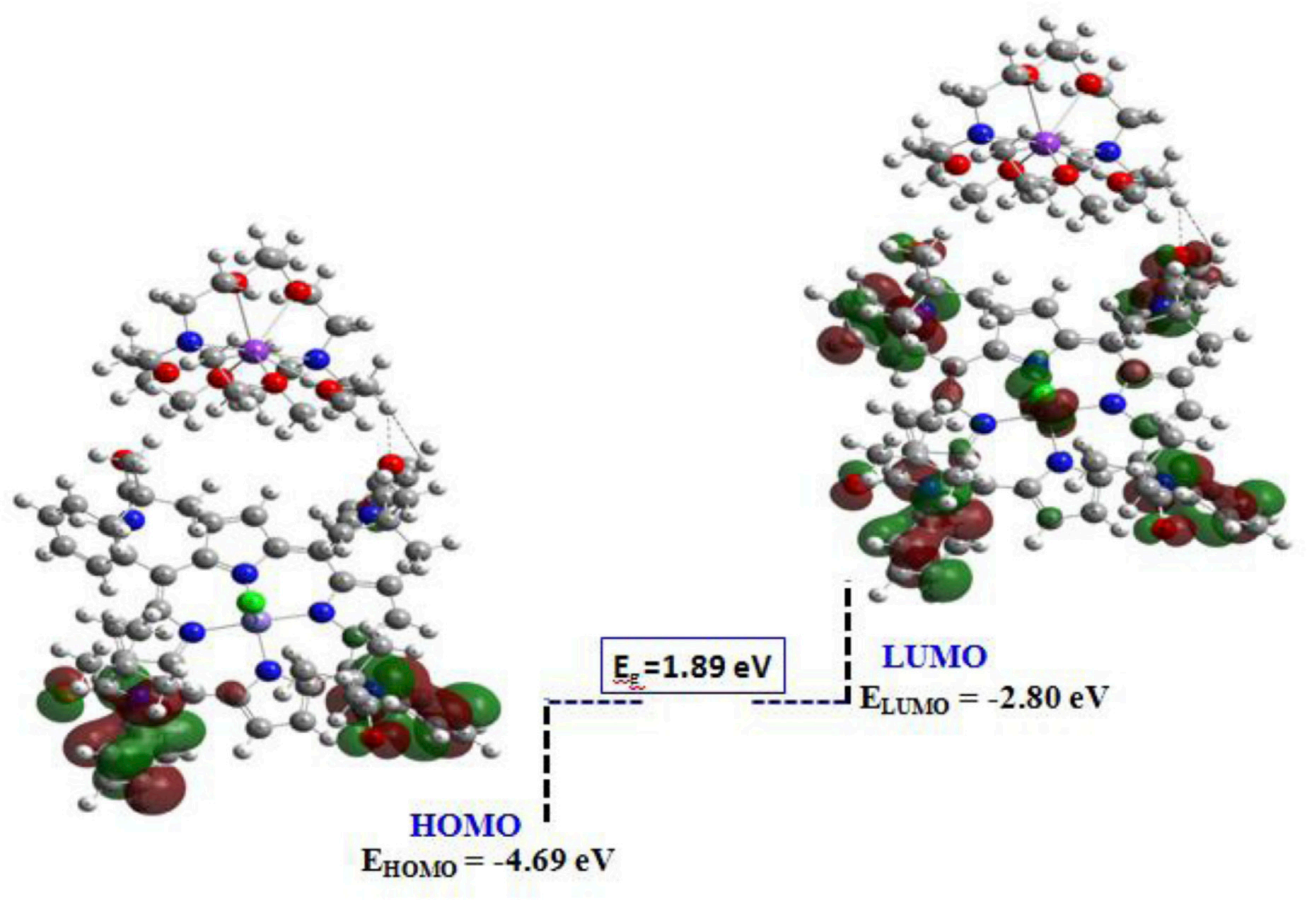
HOMO/LUMO isosurface of the current compound.

The iso-surfaces of the highest occupied molecular orbital (HOMO) and the lowest unoccupied molecular orbital (LUMO) serve as a sophisticated analytical tool for elucidating electron repartition across the material’s surface and for understanding the electronic charge transfer process (Chérif et al., 2024a; Zainab et al., 2023). Moreover, the HOMO and LUMO orbitals are beneficial to elucidate the donor and acceptor groups constituting our complex, thereby enhancing its application in sensor modules and nano-electronic devices. The energy differences between LUMO and HOMO, known as the energy gap, underscore the chemical and kinetic stability of the studied complex. The HOMO and LUMO iso-surfaces are illustrated in Figure 13. The analysis reveals that the HOMO orbital is predominantly localized around the N-ring in proximity to the central iron (Fe) metal atom. Subsequently, these electrons cross the forbidden band, ultimately becoming distributed in a manner that overlaps uniformly with the N-ring and the surrounding environment of the Fe atom. These findings demonstrated that there is a potential charge transfer occurring at the surface of the complex, particularly in the vicinity of the central Fe atom, which enhances the atomic organization in connection with Fe and contributes to the formation of a symmetrical and stable complex. Moreover, the negative HOMO value of −4.69 eV indicates a significant chemical stability coupled with heightened reactivity when interacting with electrophilic species. Additionally, the energy gap is measured to be approximately 1.89 eV, suggesting that our material exhibits semi-conductor properties, making it suitable for application in novel sensor technologies. Regarding this result, a large energy gap is linked to a hard and less polarizable system. Based on the quantum parameters outlined in Table 6, we observed a low hardness value of 0.94 eV and high electronegativity, suggesting a facile transition of electrons from the ground state (S_0_) to the excited state (S_1_). Furthermore, a notable electrophilicity index (**ω**) of approximately 9.02 eV indicates the complex’s readiness for chemical reactivity.

**TABLE 6:** Global reactivity parameters computed at the DFT/B3LYP-D3/LanL2DZ level of theory.

Reactivity parameters (eV)	
E_HOMO_	−4.69
E_LUMO_	−2.80
$E_{g}$	**1.89**
IP	4.69
EA	2.80
Μ	**−3.74**
χ	**3.74**
η	**0.94**
ω	**7.43**

**IP** (-E_HOMO_): ionization potential; **EA** (-E_LUMO_): electron affinity; **μ** ( $\left.-\frac{\text{IP}+\text{EA}}{2}\right)$ : chemical potential; **χ** ( −μ ) electronativity; **η** ( $\left.\frac{\text{IP}-\text{EA}}{2}\right)$ : global hardness; **ω** ( $\left.\frac{\mu^{2}}{2\eta }\right)$ : electrophilicity index.

#### 3.4.3 Molecular electrostatic potential (MEP) isosurface

**FIGURE 14:**
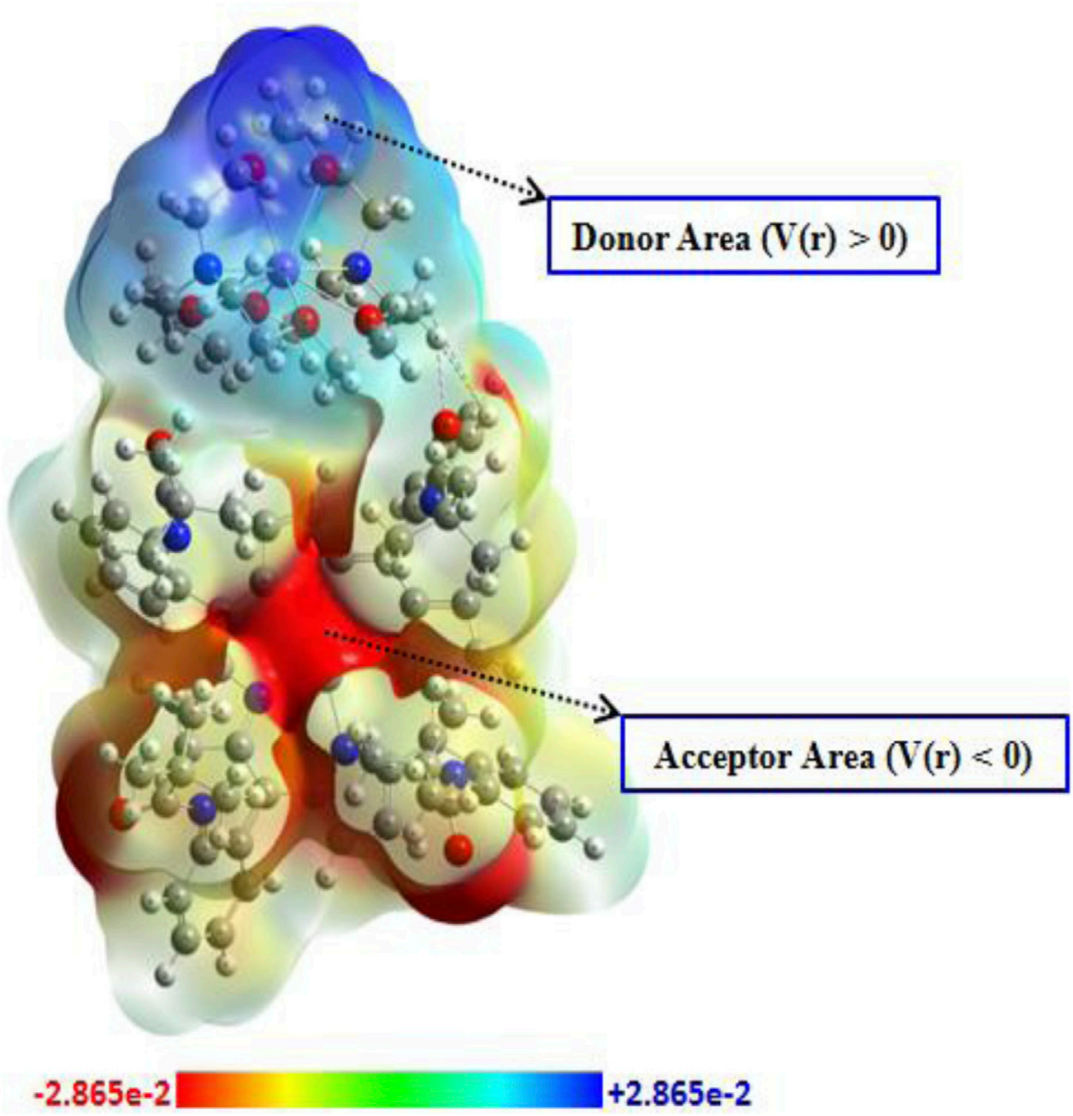
MEP plot of the studied compound.

The molecular electrostatic potential (MEP) serves as a sophisticated tool for elucidating the distribution of electron acceptor and donor regions on the surface of the studied compound and for identifying electrophilic and nucleophilic groups relevant for targeted application (Hadi et al., 2024; Hadi et al., 2023). This technique relies on the localization of the electrostatic potential (V(r)), which is represented as negative in the red regions, positive in the blue regions, and neutral in the yellow–green areas. Such insights are invaluable for evaluating sensor applications, as exemplified by our compound. The MEP plot is depicted in Figure 14. The analysis reveals that the acceptor region, associated with a nucleophilic attack, is predominantly concentrated around the central metal and the surrounding N-ring. Conversely, the donor region, indicative of an electrophilic attack, is localized around the counterion. These findings demonstrate a well-defined interaction between groups, suggesting that charge transfer may occur between the complex and counter-ion, thereby enhancing the stability and the organization of atomic groups. Furthermore, the presence of a red region within the complex facilitates interactions with neighboring molecules through electrostatic forces, further stabilizing the compound within the crystal lattice.

#### 3.4.4 Topological QTAIM analyses

To enhance our understanding of the interactions formed between atoms/groups that constitute our complex, we employed non-covalent interaction (NCI) analysis and the reduced density gradient (RDG) function (Missaoui et al., 2025; Pritha et al., 2024). The NCI tool is highly beneficial for visualizing the interactions responsible for the stability of the compound through color coding. Specifically, blue indicates hydrogen bonding interaction, green represents van der Waals forces, and red signifies the presence of steric effects (SEs). The RDG tool illustrates these forces using the same color coding in a diagram divided into the three regions (a), (b), and (c), as shown in Figure 14, which relates to the RDG as a function of signλ2×ρ (a.u.). This method is calculated using the following equation (Johnson et al., 2010):

$$\text{RDG}=\frac{1}{{2(2\left({3\pi }^{2}\right)}^{1/3}}\frac{\nabla \rho \left(r\right)}{{\left(\rho \left(r\right)\right)}^{4/3}}.$$

**FIGURE 15:**
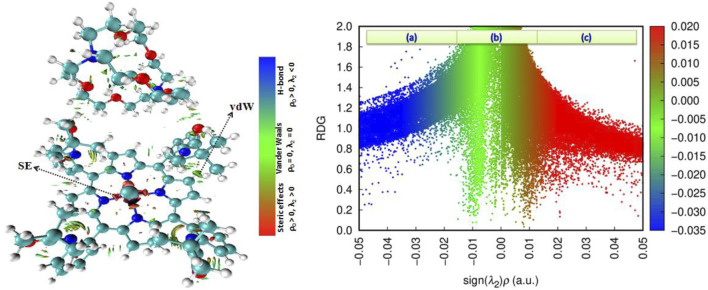
NCI and RDG iso-surface of the studied compound.

Figure 15 illustrates that the complex is primarily stabilized by van der Waals interactions, as denoted by the green color in each binding site. Additionally, the counterion stabilized in interactions with the Fe-porphyrin through electrostatic interactions (vdWs). The RDG iso-surface corroborates this finding, revealing a green peak at approximately −0.015 a.u., which suggests that the stability of the complex is predominantly governed by vdW forces. Notably, the steric effects are observed surrounding the central iron (Fe) atom.

#### 3.4.5 ELF and LOL analyses

**FIGURE 16:**
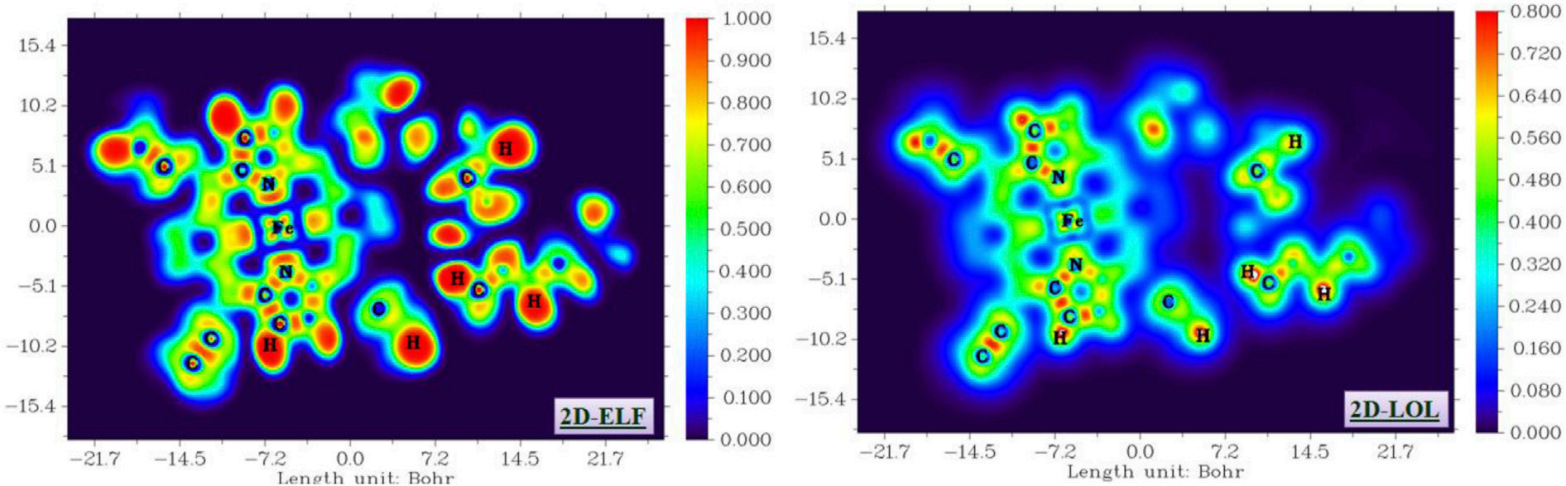
ELF and LOL isosurface of the studied compound.

The electron localization function (ELF) and localization of orbital (LOL) isosurfaces are sophisticated analytical tools for elucidating the presence of localized bonding, non-bonding, and lone pair electrons, thereby facilitating the investigation of electronic charge transfer processes within the studied compound (Chérif et al., 2024b; Belgacem et al., 2024). The ELF is derived from the density of electron pairs, and it is fundamentally based on kinetic energy, while LOL is associated with the gradient localized orbital. The ELF values range from 0 to 1, whereas the LOL values typically span from 0 to 0.8. An ELF value exceeding 0.5 indicates the presence of both bonding and non-bonding electrons, while a value below 0.5 characterizes the presence of delocalized electrons. The LOL isosurface further illustrates the delocalization of electrons within the surface of the material under study. The 2D-ELF and 2D-LOL isosurfaces are depicted in Figure 16. From the 2D-ELF image, a red color is observed surrounding H atoms, while a blue color is noted for overlapping C atoms. This is further corroborated by the LOL plot, which demonstrates a high localization of both bonding and non-bonding electrons along with delocalization in these regions. Additionally, distinct areas of electron depletion are present between the valence and inner shell. These observations suggest the presence of significant electronic charge transfer, which is advantageous for the formation of electrostatic interaction among the groups constituting the complex, thereby enhancing the stability of our compound. This conclusion is further supported by FMO and MEP analysis.

## 4 Conclusion

The synthesis and characterization of the anionic iron(II) chloro-porphyrin derivative, [K (2,2,2-crypt)][Fe^II^(TpivPP)Cl]·C_6_H_5_Cl (**I**), is described. The UV/Vis spectroscopy and the X-ray molecular structure of complex **I** indicate that this ferrous porphyrinic species is high-spin (S = 2) and presents the (d_*xy*_)^2^ (d_*xz*_)^1^ (d_*yz*_)^1^ (d_*z*_^2^)^1^ (d_*x*_^2^_-*y*_^2^)^1^ ground-state electronic configuration. The X-ray molecular structure of our chloride iron(II) picket fence porphyrin shows that the crystal lattice of complex **I** is stabilized by nonconventional C–H…O, C–H…N, and C–H….Cg (Cg is the centroid of a phenyl or a pyrrole ring) hydrogen bonds. In this paper, DFT, MEP, and NCI-RDG isosurface analyses were used to investigate the various features of complex I. The HOMO and LUMO isosurface analysis show that there is a charge transfer occurring at the surface of the complex, particularly in the vicinity of the iron(II), which contributes to the stability of complex **I**. The MEP analysis shows that the acceptor region is predominantly concentrated around the Fe(II) central ion and the surrounding N-ring. The donor region is localized around the counterion. These findings demonstrate a well-defined interaction between the [Fe^II^(TpivPP)Cl]^-^ and the [K (cryp-222)]^+^ ions, suggesting that charge transfer may occur between the complex and counterion, thereby enhancing the stability of our ferrous porphyrinic species. The NCI-RDG isosurfaces elucidate the types and natures of intermolecular interactions between the [Fe^II^(TpivPP)Cl)]^+^ and [K (crypt-222)]+ ions, which are mostly van der Waals’ type. The ELF and LOL iso-surface study supports the results obtained by MEP calculations.

## Data Availability

The original contributions presented in the study are included in the article/Supplementary Material further inquiries can be directed to the corresponding author.
