# Supplementary material for: Synthesis, X-ray crystallography, spectroscopic characterizations, and density functional theory of the chloride-bound five-coordinate high-spin Iron(II) “Picket Fence” porphyrin complex
Source: Front Chem. 2025 Jul 14;13:1607585. doi: 10.3389/fchem.2025.1607585 (PMC12302510; doi:10.3389/fchem.2025.1607585)
Supplement: Supplementary file 1 [file Supplementaryfile1.doc]

**Synthesis, X-ray Crystallography, Spectroscopic Characterizations and Density Functional Theory of the Chloride-bound Five-coordinate High-spin Iron(II) “Picket Fence” Porphyrin Complex**

Feriel Salhi ^[a]^, Mondher Dhifet ^[a, b]^, Bouzid Gassoumi ^[c] *^, Noureddine Issaoui ^[d, e]^ & Habib Nasri ^[a]^

[a] *Laboratory of Physical Chemistry of Materials (LR01ES19), Faculty of Sciences of Monastir, Avenue of the Environment, 5019 Monastir, Tunisia.*

[b] *University of Gafsa, Faculty of Sciences of Gafsa, Sidi Ahmed Zarrouk, 2112 Gafsa, Tunisia.*

[c] *Laboratory of Advanced Materials and Interfaces (LIMA), University of Monastir, Faculty of Sciences of Monastir, Avenue of Environment, 5019 Monastir, Tunisia.*

[d] *University of Monastir, Laboratory of Quantum and Statistical Physics LR18ES18, Faculty of Sciences of Monastir, Monastir, 5079, Tunisia.*

[e] *Higher Institute of Computer Sciences and Mathematics of Monastir, University of Monastir, Monastir 5000, Tunisia.*

^*^E-mail : [gassoumibouzid2016@gmail.com](mailto:gassoumibouzid2016@gmail.com)

**Contents**

1. Synthesis of the picket fence porphyrin (H_2_TpivPP) ………………………………………….2

2. UV/Vis spectroscopy…………………………………………………….…………………….3

3. IR spectroscopy………………………………………………………….…………………….3

4. X-ray molecular structure of complex **I**….…………………………….………….........…......4

**1. Synthesis of the picket fence porphyrin (H_2_TpivPP)**

**
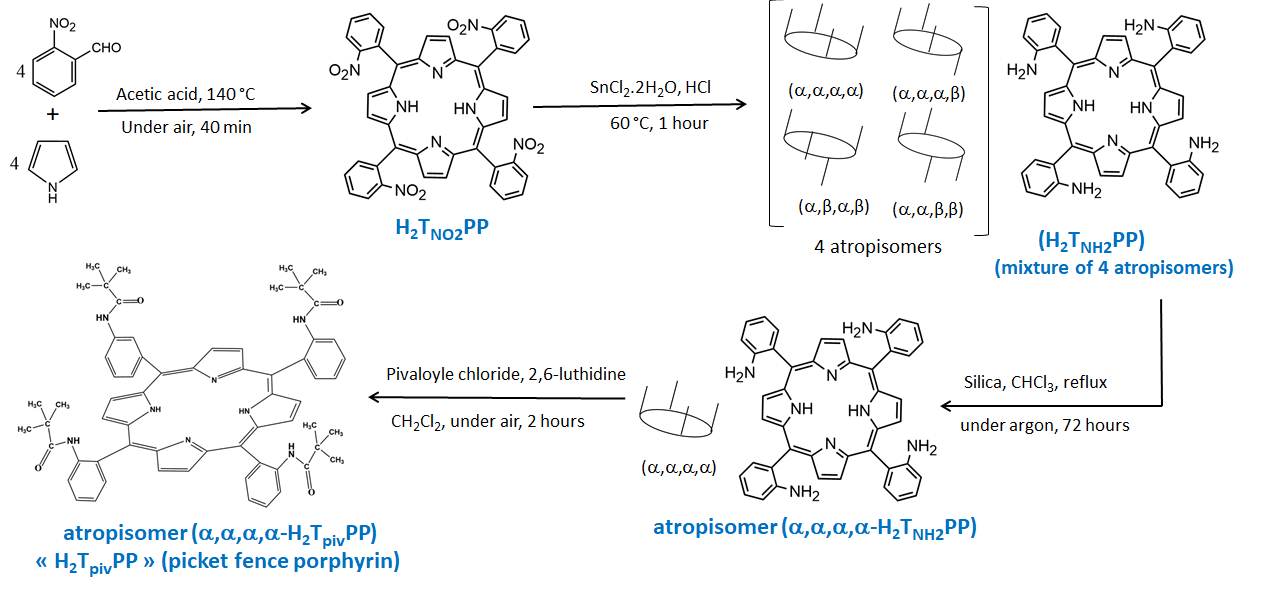
**

Scheme S1. Scheme of the preparation of the picket fence porphyrin (H_2_TpivPP).


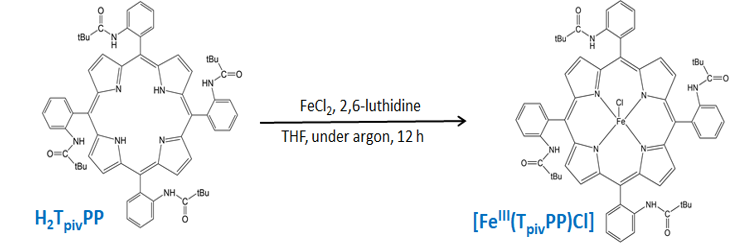


Scheme S2. Scheme of the preparation of the [Fe^III^(TpivPP)Cl].

**
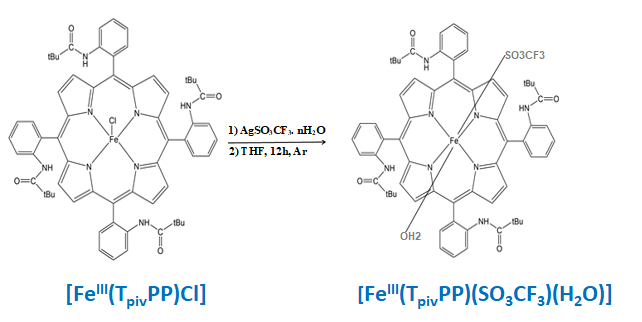
**

Scheme S3. Scheme of the preparation of the [Fe^III^(TpivPP)(SO_3_CF_3_)(H_2_O)].

**2. UV/Vis spectroscopy**


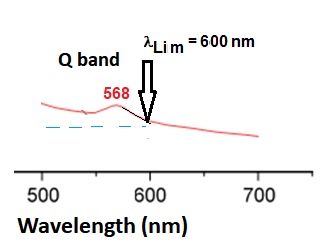


Figure S1**.** Determination of the λ_Lim_ of complex **I**

**3. IR spectroscopy**


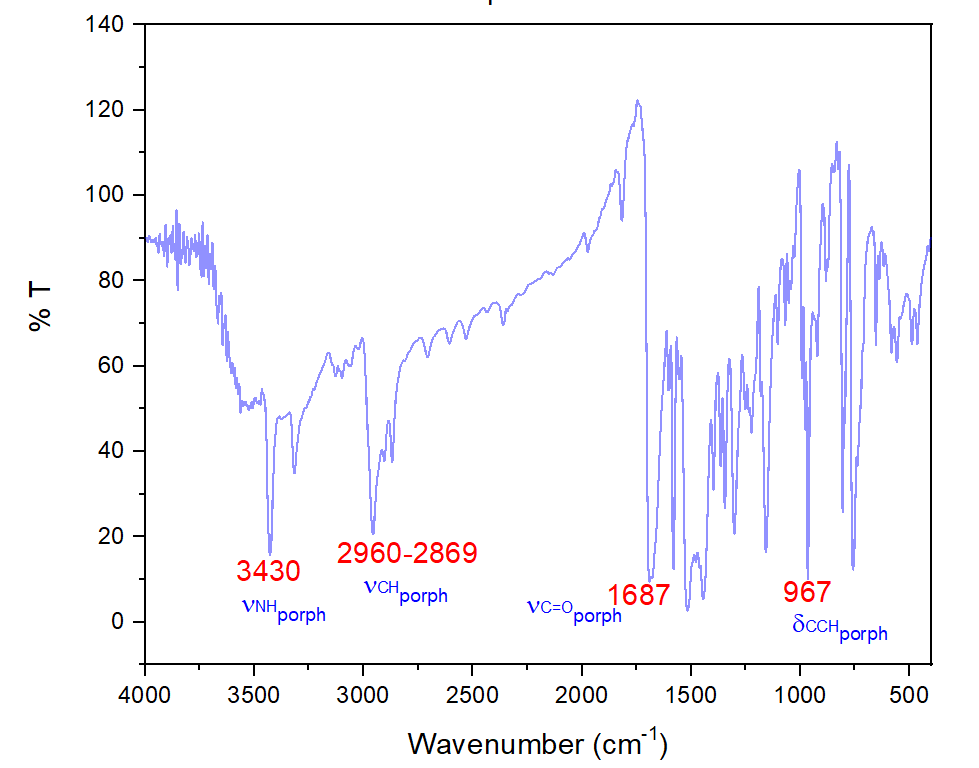


Figure S2**.** Experimental IR spectrum of H_2_TpivPP.


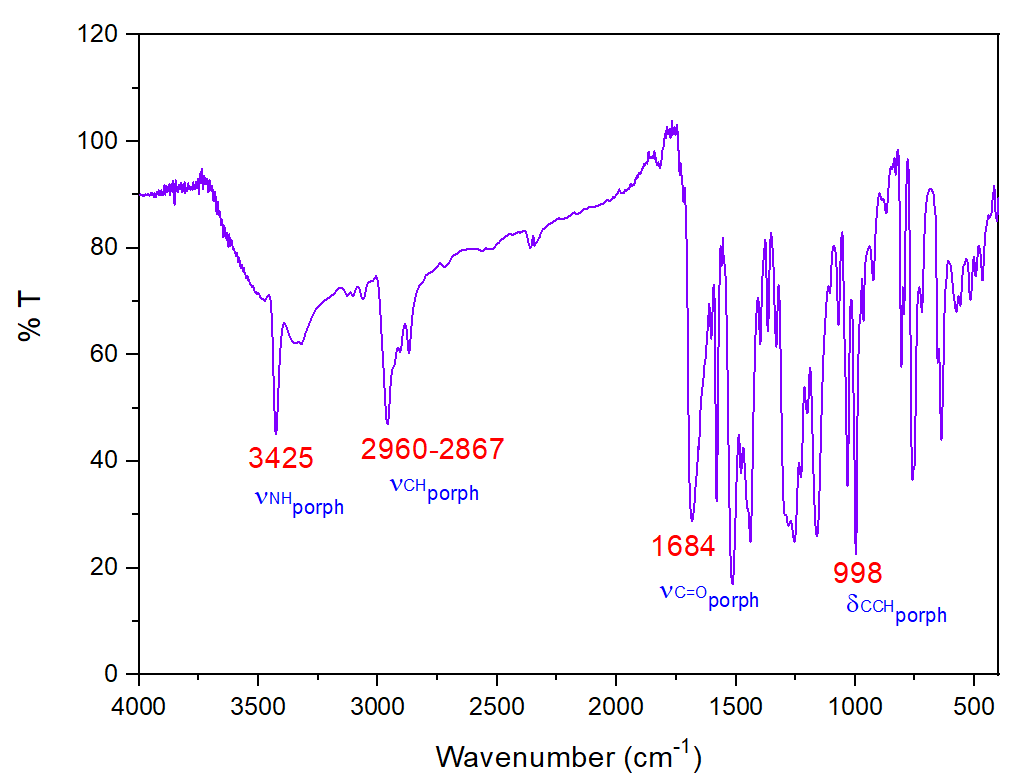


Figure S3. Experimental IR spectrum of [Fe^III^(TpivPP)(SO_3_CF_3_)(H_2_O)].

**4. X-ray molecular structure of complex I**


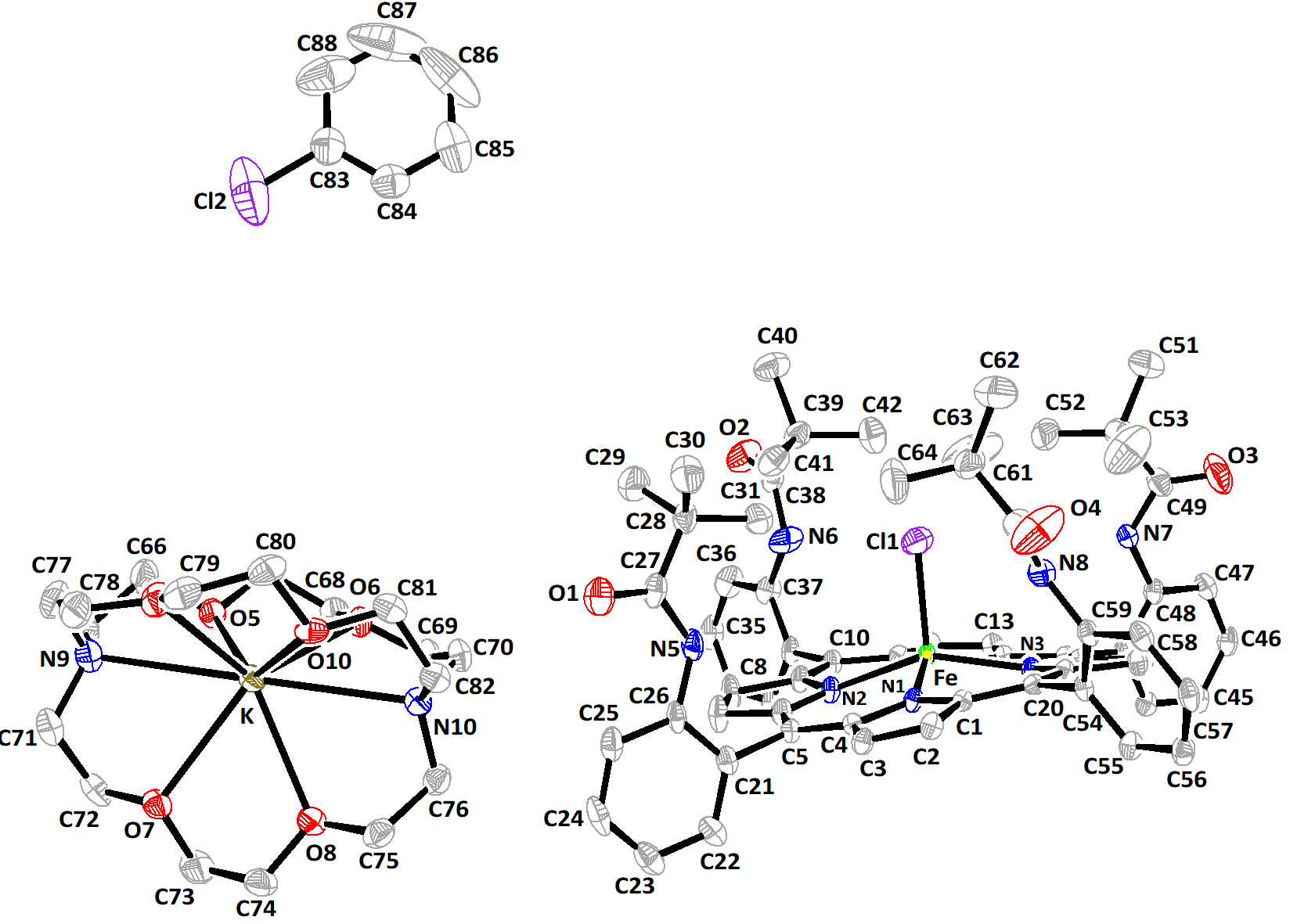


Figure S4. Diagram Ortep of the [K(crypt-222)][FeII(TpivPP)Cl]^•^C_6_H_5_Cl (**I**). Thermal ellipsoids are drawn at the 30% probability level. Hydrogen atoms have been omitted for clarity.

**
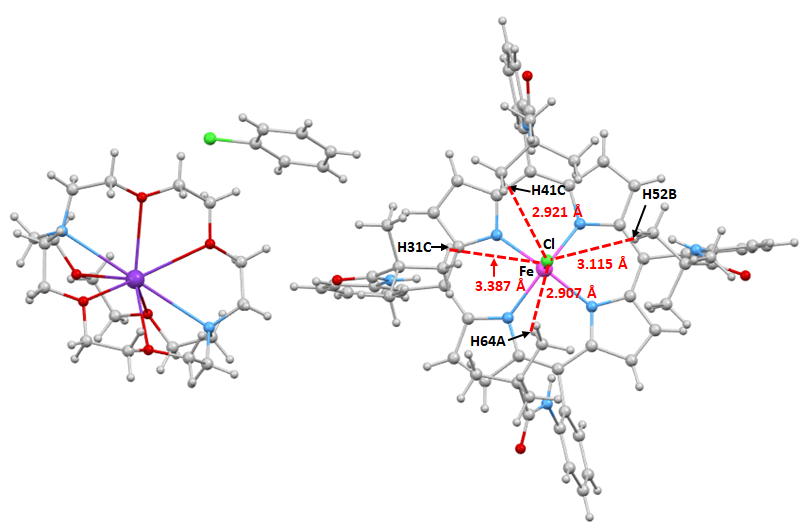
**

Figure S5. Representation showing the distances between the chloride axial ligand and the closest hydrogen atoms of the tert-butyl groups of the four pivaloyl groups of the TpivPP porphyriniate.

Table S1. Selected intermolecular interactions for complex **I**.

____________________________________________________________________________

D –H.…A**^a^** Symmetry of A D.…A (Å) D–H…A (°)

____________________________________________________________________________

C24-H24…O2 -1/2+x,1/2-y,-1/2+z 3.357(5) 172

C73-H73B…O4 1-x,-y,1-z 3.506(6) 157

C76-H76B…O2 -1/2+x,1/2-y,-1/2+z 3.161(5) 147

C80-H80A…N4 -1+x,y,z 3.368(5) 138

C34-H34…Cg11**^b^** 5/2-x,-1/2+y,3/2-z 3.783(4) 165

C57-H57…Cg11**^b^** 5/2-x,-1/2+y,3/2-z 3.738(4) 146

C66-H66B…Cg3**^c^** -1+x,y,z 3.894(4) 151

C71-H71B…Cg12**^d^** 1-x,-y,1-z 3.632(4) 133

C79-H79B…Cg1**^f^** -1+x,y,z 3.957(4) 176

C80-H80A…Cg4**^h^** -1+x,y,z 3.387(4) 136

C82-H82B…Cg9**^i^** 1-x,-y,1-z 3.625(4) 137

__________________________________________________________________________

**^a^** D = donor atom and A = acceptor atom.

**^b^** Cg11 = Centroid of the phenyl ring : C43 --> C44 --> C45 --> C46 --> C47 --> C48.

**^c^** Cg3 = Centroid of the pyrrole ring : N3 --> C11 --> C12 --> C13 --> C14.

**^d^** Cg12 = Centroid of the phenyl ring : C54 --> C55 --> C56 --> C57 --> C58 --> C59.

**^f^** Cg1 = Centroid of the pyrrole ring : N1 --> C1 --> C2 --> C3 --> C4.

**^h^** Cg4 = Centroid of the pyrrole ring : N4 --> C16 --> C17 --> C18 --> C19.

**^i^** Cg9 = Centroid of the phenyl ring : C21 --> C22 --> C23 --> C24 --> C25 --> C26.

Table S2. Hydrogen bonds with H…A < r(A) + 2.000 Å and <DHA > 110 ° for complex **I.**

____________________________________________________________________________

D-H d(H...A) (Å) D–H…A (°) d(D...A) (Å) A Symmetry of A

____________________________________________________________________________

C25-H25 2.279 120.60 2.881 O1

C36-H36 2.238 120.54 2.841 O2

C47-H47 2.407 111.67 2.896 O3

C58-H58 2.281 115.15 2.819 O4

C73-H73B 2.575 157.14 3.508 O4 [-x+1, -y, -z+1]

C76-H76B 2.286 146.61 3.160 O2 [x-1/2, -y+1/2, z-1/2]

C80-H80A 2.567 137.99 3.368 N4 [x-1, y, z]

____________________________________________________________________________
